# Assessment of Dietary Intake of Iodine and Risk of Iodine Deficiency in Children with Classical Galactosaemia on Dietary Treatment

**DOI:** 10.3390/nu15020407

**Published:** 2023-01-13

**Authors:** Dearbhla Milner, Fiona Boyle, Jenny McNulty, Ina Knerr

**Affiliations:** 1 School of Public Health, Physiotherapy and Sports Science, University College Dublin, Belfield, D04 V2P1 Dublin, Ireland; 2 Department of Nutrition and Dietetics, National Centre for Inherited Metabolic Disorders, Children’s Health Ireland, Temple Street, D01 XD99 Dublin, Ireland; 3 National Centre for Inherited Metabolic Disorders, Children’s Health Ireland, Temple Street, D01 XD99 Dublin, Ireland

**Keywords:** iodine, iodine deficiency disorders, galactosaemia, diet, inborn error of metabolism, rare disease, thyroid function

## Abstract

Iodine is an essential mineral required for the synthesis of thyroid hormones. Iodine plays a critical role in growth and neurocognitive development. Classical galactosaemia is a disorder resulting from an inborn error in galactose metabolism. Its current management consists of life-long lactose and galactose dietary restriction. This study estimated dietary intakes of iodine in infants and children with classical galactosaemia in the Republic of Ireland. The diets of 43 participants (aged 7 months–18 years) with classical galactosaemia were assessed for iodine intake using an iodine-specific food frequency questionnaire. Intakes were compared to the European Food Safety Authority (EFSA) dietary recommendations for iodine intake. The potential role of iodine fortification of dairy alternative products was also examined. There were no significant differences observed between sex, ethnicity and parental education and meeting dietary iodine recommendations. Differences, however, were seen between age groups, causing the p value to approach statistical significance (*p* = 0.06). Infants consuming infant formula were likely to meet iodine recommendations. However, over half (53%) of children aged 1–18 years had average intakes below the recommendations for age. For these children, consumption of iodine-fortified dairy alternative milk was the leading source of iodine in the diets, followed by fish/shellfish and eggs. An assessment of iodine intake should be undertaken during dietetic reviews for those with classical galactosaemia. Mandatory iodine fortification of all dairy alternative products would result in 92% of the total population cohort meeting iodine recommendations based on their current consumption.

## 1. Introduction

Iodine is an essential micronutrient, which is required by the thyroid gland to produce the thyroid hormones triiodothyronine (T3) and thyroxine (T4) [1]. T3 and T4 control a variety of important physiological processes including growth, neurological development, metabolic regulation and reproductive function [1,2]. Iodine deficiency results in a wide range of health consequences [3] (Table 1) and has been identified as the single greatest cause of preventable cognitive impairment worldwide [3].

Rich dietary sources of iodine include milk and milk products, fish/shellfish (particularly white fish), seaweed and eggs. Due to the exclusion of many of these foods, vegans are at increased risk of iodine deficiency [4]. 

Classical Galactosaemia (CG) is an inherited disorder of galactose metabolism that results from a severe deficiency of the galactose-1-phosphate-uridyltransferase (GALT) enzyme [5]. The prevalence of CG worldwide is 1:16,000–60,000 live births [6]. The incidence of CG in the Republic of Ireland is approximately 1:16,500 [7]. This is reflective of a high incidence of 1:450 births of CG in the Irish Traveller population [8]. This disorder impairs the body’s ability to break down galactose. Galactose is a constituent of lactose, which is the primary sugar in human milk, cow’s milk, all other mammalian milks and the majority of infant formulas. Ingestion of lactose (and therefore galactose) in those with CG leads to the accumulation of galactitol, galactonate and galactose-1-phosphate in abnormal quantities. The measurement of galactitol, galactonate and erythrocyte galatose-1-phosphate (Gal-1-P) has been investigated for the biochemical monitoring of patients with CG and correlated with individual galactose intake [9]. Gal-1-P, in particular, is a toxic metabolite which leads to a lack of feedback inhibition of the enzyme galactokinase and, in the absence of glucose, also leads to energy deficiency in the cell [10]. If CG is left untreated, poor feeding, weight loss, vomiting, diarrhea, hypotonia, cataracts, jaundice, hepatic failure and coagulopathy, renal tubular disease, E-coli sepsis and death can occur [5]. A life-long lactose- and-galactose restricted diet is currently recommended for management of CG [5]. Those affected are recommended to exclude milk and milk products from mammalian sources. Mature cheese with a galactose content of less than 25 g/100 g is permitted in CG [5]. 

Similarly to vegans, those with CG may be considered at increased risk of inadequate iodine intake. There are currently no studies published investigating iodine intake among infants and children with CG. 

The aims of the current study were to estimate dietary iodine intake among infants and children with CG and compare intakes to recommendations by the European Food Safety Authority (EFSA) [11]. The study tested the hypothesis that Irish children with CG are at a higher risk of iodine insufficiency compared to children in the general Irish population. The potential role of iodine fortification of all dairy alternative products was also examined.

## 2. Materials and Methods

### 2.1. Study Participants

The patient cohort included all those with CG under or equal to 18 years of age attending services in the National Centre for Inherited Metabolic Disorders in Children’s Health Ireland (CHI), Temple Street, Dublin, Ireland, between November 2021 and April 2022. The sample population consisted of 91 patients. A total of 43 patients were enrolled in the study, resulting in a 47% participation rate.

Ethical approval was not required as the data collected was non-sensitive information that was gathered during a patient’s routine clinical consultation. A clinical audit application form was submitted to and approved by the research department in CHI.

### 2.2. Study Design

#### 2.2.1. Demographics

Demographic parameters were collected on the participants’ sex, date of birth, ethnicity/cultural background (Irish/Irish Traveller or other) and the highest level of education obtained by the parent/guardian.

#### 2.2.2. Assessment of Estimated Iodine Intake

A 58-item semi-quantitative iodine-specific food frequency questionnaire (I-FFQ) was designed to assess the iodine intake of infants and children with CG (see Supplementary Material Document S1). This was based on two validated FFQs: an FFQ used by Safefood to assess dietary iodine intake in Ireland [12] and another used as part of an Australian study examining the dietary iodine intake of pregnant women [13]. A reference portion size food atlas was developed (see Supplementary Material Document S2) for use with the I-FFQ to establish the typical portion size of food items consumed. It was based on a standardized food atlas, ‘Carbs and Cals’ [14], for portion sizes and the ‘McCance and Widdowson composition of foods integrated dataset’ (MWFID), for the iodine content of foods [15]. A local database of the reported iodine content of dairy-free alternatives and vitamin and mineral supplements was also used. 

The I-FFQ reviewed the frequency, quantity and type of food eaten in recent months. It included the following food categories: fish/shellfish, dairy alternative products, permitted cheeses, eggs, mixed dishes (for example, pizza, quiche and fresh pasta), other foods (sushi, seaweed, cashew nuts) and lactose-containing foods (dairy milk and milk products). The frequency categories included: never or less than once a month, once a month, once in two weeks, once a week, 2–3 times a week, 4–6 times a week, once a day and more than once a day. Additional questions were included on whether the patients were taking an iodine-containing supplement (including seaweed or kelp supplements) and the use of iodized salt. The questionnaire was administered as an interview with the participants’ guardian (or participant if appropriate). 

The reported amount of iodine consumed was divided by the frequency of consumption to obtain an average daily iodine intake from each food group in micrograms (µg) and, subsequently, a total average daily iodine intake for each participant (µg/day).

#### 2.2.3. Assessment of Estimated Iodine Intake versus Recommendations

The estimated average iodine intake of individuals was compared to the adequate intake (AI) values for iodine intake recommended by the European Food Safety Authority (EFSA) [11]. Their average daily iodine intake was also compared to the tolerable upper level (UL) for their age [16]. Subjects were split into the five EFSA adequate intake age (AI) groups. ‘Adequate intake’ was classified as equal to or greater than EFSA recommendations. Conversely, ‘Inadequate intake’ was classified as less than the EFSA recommendations. 

#### 2.2.4. Comparison of Iodine Intake in Classical Galactosaemia versus the General Population

Iodine intake and levels of adequacy in this CG cohort were compared to data available for the Irish general population from a recent study of iodine intakes among Irish children [17]. 

#### 2.2.5. Projected Impact of Iodine Fortification on Dairy Alternative Products

A projected calculation was applied to investigate whether fortification of dairy alternative products with iodine would allow those with CG to achieve the iodine recommendation for their age group. These estimations were based on iodine fortification of dairy alternative products as follows: non-iodine fortified milk to 22.5 µg/100 mL (equivalent to the iodine content of leading brands of iodine fortified milks in Ireland), yoghurts to 34 µg/100 g, custards to 28 µg/100 g (equivalent to dairy forms as per MWFID) and cheese to 11–22 to µg/100 g (equivalent to dairy forms dependent on the type of cheese consumed as per MWFID). These values were divided by the frequency of their consumption in order to predict an average projected total iodine intake per day. This hypothetical iodine intake was compared with AI and UL values. 

### 2.3. Statistical Analysis

Following the completion of data collection, the dataset was imported from Microsoft Excel to IBM SPSS Statistics Version 27 for analysis. Descriptive statistics were performed, and the results were reported as absolute values and percentages. Due to low numbers, subjects were then split into three age groups to investigate differences between infants (7–11 months), children (1–10 years) and adolescents (11–18 years) in a subsequent analysis examining impact of sex, ethnicity or cultural background and education level on nutrient adequacy levels. Chi-square tests with Phi Crammer’s V were performed to investigate significant differences between demographics. A *p* value of < 0.05 was accepted as statistically significant. Non-parametric tests were performed throughout since the data was not normally distributed. Total iodine intakes (μg/day) were expressed as median and interquartile ranges. Comparisons of average iodine intakes between and within groups were performed using one-way ANOVA. A Spearman correlation test was used to assess the relationship between estimated iodine intakes and iodine intake from iodine-fortified dairy alternative milk, fish/shellfish and eggs. These correlations were interpreted according to Cohen’s correlation guidelines [18].

## 3. Results

### 3.1. Demographics

Table 2 summarizes the general characteristics of the participants. Out of the 43 participants, 60.5% were female and 39.5% were male. The ages of the cohort ranged from 7 months to 18 years of age, with a median age of 6 years ± 5.04. Over half of the study group was Irish Traveller (51.2%).

### 3.2. Assessment of Estimated Iodine Intake in Age Groups

**Figure 1:**
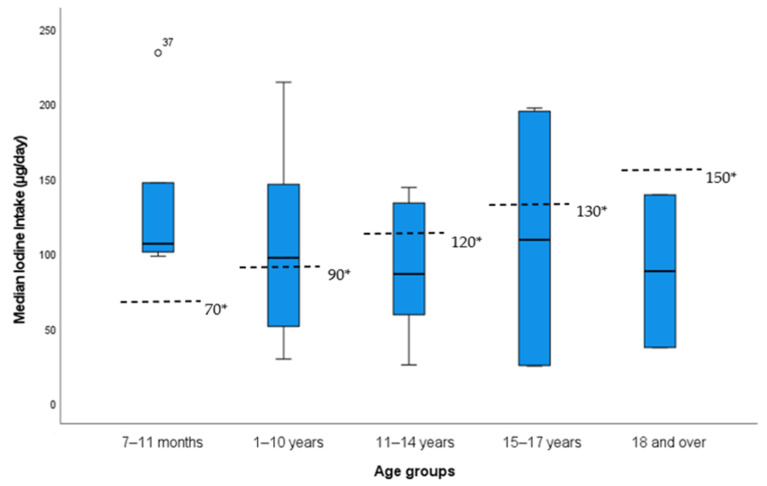
Distribution of estimated iodine intake across EFSA AI age groups. * EFSA adequate intake value for age [11].

The median iodine intake varied between 106 µg/day in the 7–11 months age group, 97 µg/day in the 1–10 years age group, 86 µg/day in the 11–14 years age group and 109 and 88 µg/day in the 15–17 years and 18 years age groups, respectively. Two patients exceeded the daily UL for iodine for their age. The distributions of iodine intakes per day across the age groups are shown in Figure 1. The EFSA AI for age [11] is included for comparison.

### 3.3. Assessment of Estimated Iodine Intake versus Recommendations

**Figure 2:**
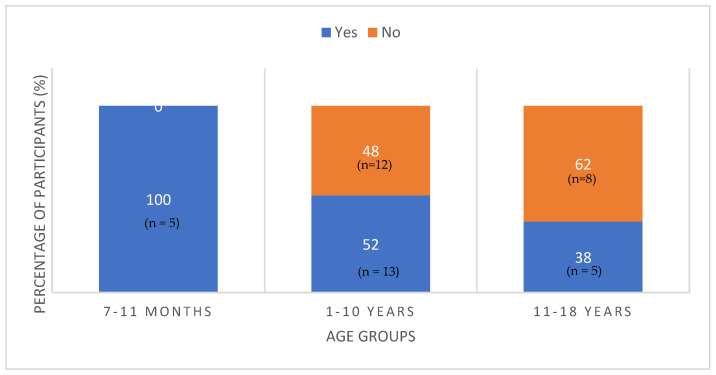
Percentage of participants meeting EFSA AI recommendations for iodine by age groups.

Forty seven percent of the total population had an iodine intake below the EFSA recommended AI values for their age. The proportion of patients meeting the EFSA AI values by age groups is illustrated in Figure 2. All infants less than 1 year achieved the recommended intake for their age. In contrast, 62% of children aged 11–18 years fell below the recommended intake. This explains why the overall p value between the three age groups and meeting EFSA AI values approached significance (*p* = 0.06). 

A chi-square test for independence indicated no significant association between sex and meeting the EFSA AI value for iodine (*p* = 0.71). Similarly, there was no significant difference found between either the ethnic/cultural groups or education level of the guardian and iodine adequacy. 

### 3.4. Comparison of Iodine Intake in CG versus the General Population

Our findings from our food frequency questionnaire revealed that 59% of children with CG in this study aged 5–12 years (n = 17) did not meet the EFSA AI values for iodine. In comparison, Kane et al. assessed the iodine intake of Irish children 5–12 years using a four-day weighed food diary. They reported that 43% of Irish children aged 5–12 years (n = 600) did not meet the EFSA AI values [18]. In our cohort, as stated, the median (interquartile range) iodine intake for children with CG aged 5–12 years was 77 μg/day (48, 129 μg/day). The same study reported that the median iodine intake for children in the general population aged 5–12 years was 107 μg/day (69, 164 μg/day) [15]. Data on iodine intake was not available for the other age categories for comparison. 

### 3.5. Dietary Sources of Iodine for Children with CG

**Figure 3:**
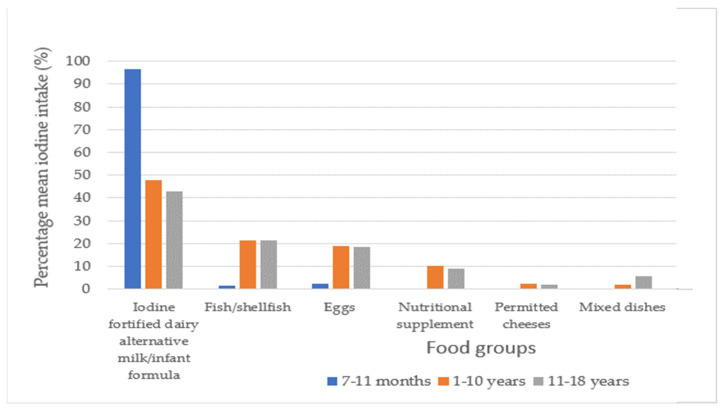
Dietary sources of iodine for infants and children with classical galactosaemia.

Figure 3 illustrates the main dietary sources of iodine for each age group as a percentage of total iodine intake. For infants aged 7–11 months, infant formula contributed to 96% of the iodine intake. For children 1–10 years, iodine-fortified dairy alternative milk contributed to 48% of the iodine intake. Similarly, for those aged 11–18 years, 43% of the iodine intake was obtained from an iodine-fortified dairy alternative milk. Twenty one percent of children aged 1–18 years consumed a nutritional supplement containing iodine. Sixty three percent of children aged 1–18 years did not meet the EFSA AI values for iodine through diet alone. No iodized salt consumption was reported.

**Figure 4:**
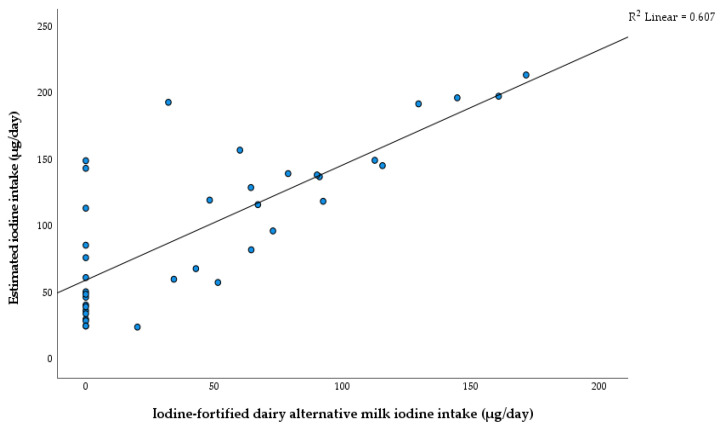
Correlation between estimated iodine intake (µg/day) and iodine-fortified dairy alternative milk iodine intake (µg/day) for children aged 1–18 years.

A positive relationship between iodine intake from iodine-fortified dairy alternative milk (µg/day) and estimated iodine intakes (µg/day) was seen (R^2^ = 0.61, Figure 4). The same analysis examining the relationships between iodine intake from fish/shellfish and eggs (µg/day) and estimated iodine intakes (µg/day) demonstrated a weak association.

### 3.6. Projected Impact of Iodine Fortification on Dairy Alternative Products in Ireland

At the time of our study, only 19 of the 178 dairy alternative milks reviewed on the market in Ireland were fortified with iodine. The reported levels of fortification ranged from 13 µg to 56 µg per 100 ml. None of the dairy alternatives for yoghurts, custards/desserts and cheeses were fortified with iodine. Two strategies to examine the impact of further iodine fortification were modelled. Strategy 1 examined the projected impact of substitution of non-iodine fortified dairy alternative milk with an iodine-fortified dairy alternative milk (22.5 µg/100 mL) on meeting the EFSA recommendations for iodine [11]. This strategy would result in an increase from 47% to 71% of children aged 1–18 years achieving an iodine intake above the EFSA AI values (Table 3). This level of fortification would not result in any further participants exceeding the UL for age. 

Strategy 2 examined the projected impact of iodine-fortified dairy alternative milk substitution and fortification of dairy alternative yoghurts, custard/desserts and cheeses on meeting the EFSA recommendations for iodine. Our results illustrated that further iodine fortification of dairy alternative milk, yoghurts, custard/desserts and cheese to the levels described in the methods section would result in 92% of children aged 1–18 years achieving an iodine intake above the EFSA AI values. The number of children exceeding the UL would increase from two to five.

## 4. Discussion

Iodine is a critical nutrient for the healthy functioning of the thyroid gland [1,3]. It is of particular importance during infancy and childhood [3]. The European Food Safety Authority (EFSA) report on iodine established a cause-and-effect relationship between an adequate dietary intake of iodine and its contribution to normal cognitive development [11]. Iodine’s role in the growth and development of infants and children was also acknowledged [11]. Children with biochemical evidence of iodine deficiency experience a higher level of oxidative stress than their healthy peers with iodine sufficiency [19]. Conversely, iodine in excess is much less harmful as it may only affect a small percentage of sensitive individuals, whereas iodine deficiency can affect an entire cohort or parts of a population with endemic consequences [20]. 

In Ireland, milk and other dairy products tend to be the main sources of dietary iodine intake [12]. Due to the recommended exclusion of mammalian milks and their products, those with CG may be considered at risk of iodine insufficiency. 

In spite of early dietary treatment and management, significant long-term complications in CG are still common [5]. These may include cognitive impairment, speech and/or language difficulties, primary ovary dysfunction, reduced bone density and psychiatric complications [5]. The proposed mechanisms for the pathophysiology of CG include the accumulation of galactose metabolites, impaired glycosylation, altered signalling pathways and impaired redox homeostasis [21]. In addition to thyroid health, sufficient iodine intake could have an additional beneficial effect in those with CG by reducing oxidative stress. Other factors may also increase the risk of thyroid dysfunction in children with CG. Thyroglobulin, the major glycoprotein of the thyroid, contains up to 10% carbohydrate residues, which also include galactose [22]. A lack of completion of thyroglobulin glycosylation may, therefore, be considered a potential acquired factor for thyroid dysfunction in patients with CG later in life. Thyroid disorders are often multifactorial. We here discussed new insights in paediatric patients with CG on a lactose-free galactose-restricted diet by evaluating iodine intakes in our Irish cohort. 

### 4.1. Demographics

Over half of our study population was from the Irish Traveller community. This was a representative sample of the whole cohort attending NCIMD in the Republic of Ireland. Studies have suggested that half of Irish Travellers have poor functional literacy [23]. Poor literacy can be a barrier to healthy food choices [24]. Although literacy levels were not assessed in this study, no significant differences were found between the ‘Irish’ and ‘Irish Traveller’ groups or the different education level groups and achieving dietary iodine recommendations. 

### 4.2. Assessment of Estimated iodine Intake versus Recommendations

All infants aged 7–11 months met the EFSA AI values due to the consumption of infant formula, which contains iodine. Our data, however, suggested that as requirements for iodine increase with age, it is more difficult to meet EFSA recommendations. This was demonstrated, as there was a 28% increase in patients falling below the EFSA recommendations in the 11–18-year-old age group compared to the 1–10-year-old age group. Interestingly, no significant differences were observed in meeting the requirements between the older age groups; however, this could be due to the small sample size of this study. 

There are several reasons why older children may find it more difficult to meet iodine intake recommendations. Children with CG typically transition from infant formula to a dairy alternative milk after 1 year of age. It will be challenging to meet iodine recommendations if a non-iodine-fortified dairy alternative milk is used. Even older children who are consuming an iodine fortified dairy alternative milk may have an intake below the EFSA AI recommendations because the target proves difficult to meet without consuming considerably large volumes. Ideally, patients would be consuming a varied diet with different iodine sources such as fish/shellfish and eggs, which contribute to their overall iodine intake. Children with CG would need to consume about 550 to 700 mL of iodine-fortified dairy alternative milk, depending on age, if they are solely relying on iodine-fortified dairy alternative milk (≈22.5 µg/100 mL) to meet the recommendations. Furthermore, it is common for dairy consumption to decline in children as they enter adolescence [25,26]. This observed time trend has been identified in several studies and it could be a potential reason of reduced dairy alternative milk consumption in this age group [25,26]. 

### 4.3. Comparison of Iodine Intake in CG versus the General Population

Data on iodine intake in the general Irish population is limited. Only one study examined iodine intake in children of 5–12 years of age using a four-day weighed food diary [17]. Using a food frequency questionnaire to assess iodine intake, children with CG in our study appeared to have a lower average iodine intake compared to their Irish peers. Furthermore, the higher incidence of patients with CG failing to meet the EFSA AI values demonstrates an elevated risk of iodine insufficiency among children with CG.

### 4.4. Dietary Sources of Iodine for Children with CG

At present, iodine-fortified milk plays a critical role in helping children with CG achieve the current recommendations for iodine intake. As demonstrated, a large positive correlation (R^2^ = 0.61) was observed between estimated iodine intake and iodine intake from iodine-fortified dairy alternative milk. This was similar to previous studies looking at dietary sources of iodine and iodine status [12,17]. A positive correlation was also determined between dairy milk consumption and iodine intake in a cross-sectional study examining the iodine status of UK schoolgirls [27]. This demonstrates how iodine-fortified dairy alternative milks, when used as a substitute for dairy milk, could improve iodine intakes in children with CG. No children met the EFSA AI values for age without consuming an iodine-fortified dairy alternative milk or a nutritional supplement containing iodine. This reinforces the importance of consuming iodine-fortified dairy alternative milk. 

Fish/shellfish contributed 30% of the average iodine intake in children with CG aged 5–12 years. Comparatively, fish/shellfish contributes just 2% to the iodine intake of the Irish population of 5–12-year-olds according to data published by the FSAI [28]. Generally, fish is a less popular food option and is typically not consumed every day [28]. The iodine content of fish also varies considerably [15]. White fish such as cod and haddock are particularly rich sources of iodine. Oily fish and shellfish such as salmon, tuna and prawns, conversely, contain much smaller amounts of iodine. Eggs could contribute to a significant proportion of the average iodine intakes for children with CG. 

### 4.5. Projected Impact of Iodine Fortification on Dairy Alternative Products in Ireland

A United Kingdom (UK) study in 2017 found that only three out of forty-seven dairy alternative milks analysed were fortified with iodine [29]. Our findings revealed that more brands are choosing to fortify their dairy alternative milks with iodine, as a further sixteen milks have been identified as iodine-fortified since this study was published. There are several barriers that may prevent individuals from choosing iodine-fortified dairy alternative milks, such as cost, accessibility, awareness and literacy. A study published in the UK indicated poor knowledge of iodine in pregnant women and new mothers [30] and women of childbearing age [31]. A total of 56% of women were unable to identify iodine-rich sources [29] with only 8–9% correctly identifying dairy as a source [30,31]. 

The Irish Department of Health ‘Food Pyramid’ recommends three servings for 5–8-year-olds and five servings for 9–18-year-olds from the milk, yoghurt and cheese food group daily [32]. From this food group, children with CG can only obtain iodine from a fortified dairy alternative milk or permitted cheeses. Presently, other dairy alternative products such as yoghurts, custards and cheeses are not fortified with iodine. Permitted cheese consumption was low in our CG cohort. From our results, it was evident that the substitution of dairy alternative products with an iodine-containing equivalent would have a positive impact on the iodine intake of children with CG. 

Representatives from the Food and Drink Federation, the Vegan Society and the British Dietetics Association addressed issues surrounding iodine food fortification. In May 2021, they committed to work with market leaders to ensure all dairy alternative products sold in the UK are fortified with iodine equivalent to the amounts available in cow’s milk (25–50 µg per 100 mL) [33]. The findings of our study support this plan for the iodine fortification of all dairy alternative milks and dairy alternative products.

### 4.6. Effect of Soya as a Goitrogen

Soya and soya products contain soy isoflavones, which are known to be goitrogenic. They can inhibit iodine utilisation for thyroid hormone synthesis. Studies examining the goitrogenic effect on thyroid health suggest that the effect is increased in iodine deficiency [34]. Interestingly, participants in an Irish study who consumed soya-based drinks had the lowest urinary iodine concentration, but numbers were small [12]. Furthermore, in an article by Vance, it was suggested that adding iodine to a soya based dairy alternative milk may not have the same impact as adding iodine to a beverage that is derived from a non-goitrogenic source, for example, oat milk, almond milk or rice milk [35]. This suggests that children consuming a soya-based alternative milk may not be utilising the entirety of the iodine they ingest, putting them at a higher risk of having an insufficient iodine status. In our sample, 30% of patients consumed a non-iodine-fortified soya-based alternative milk. Soya milk consumption, therefore, coupled with a low iodine intake could be cause for concern in CG. 

### 4.7. Strengths and Limitations of the Study

The strengths of this study included the fact that the population cohort was nationally representative of all infants and children with CG in Ireland. Our national centre follows one of the largest cohorts of individuals with CG internationally [7]. The sample was diverse, consisting of patients of varying ages, sex, ethnicities/cultural groups and guardians with varying educational backgrounds. The study design was favourable for several reasons. Firstly, using a food frequency questionnaire (FFQ) to assess iodine intake allowed the habitual intake of an individual to be captured. This is advantageous, as iodine intake can be extremely variable. Secondly, the nature of an FFQ is less likely to influence dietary behaviour of the patients as it is retrospective. The FFQ was administered as an interview, ensuring that no question was left unanswered and portion sizes were confirmed. 

This study had a considerable participation rate of 43%. Once stratified by age categories, however, the numbers within groups became smaller, which reduced the statistical power of the study. This increased the margin of error and made comparison with the general population more difficult. The limitations of the study design included the fact that the I-FFQ was not validated itself but was an adaption of two previously validated I-FFQs. Meat and cereal products were omitted due to their considerably low iodine content and may have led to over-reporting of the other dietary sources asked in the I-FFQ. Other limitations included potential systematic errors and biases in the estimates due to the approximation of portion sizes using a food atlas. There was also a lack of data available on the iodine content of various cheeses which are permitted in the diets of those with CG. Furthermore, the estimated iodine intake values could not be contrasted against biomarkers of iodine status, given that no biological samples were collected for this study. As a result, the findings in this study should be interpreted with caution. To provide a more comprehensive outlook of iodine status, future studies should aim to include dietary, biochemical and clinical assessments.

Our study is the first published study to assess iodine intake along with the risk of iodine deficiency in children with CG. Our findings indicated that children following a lactose and galactose restricted diet for management of CG are at an increased risk of iodine deficiency when compared with the general paediatric population.

## 5. Conclusions

The aim of this study was to investigate iodine intake in Irish infants and children with CG and to estimate the risk of insufficient iodine intake. This study suggests that infants consuming the recommended amounts of formula are likely to meet their iodine needs. In contrast, 63% of children aged 1–18 years in our cohort had suboptimal iodine intake from diet alone. This finding may have clinical implications for patients with CG, as iodine deficiency is linked to a number of adverse health consequences. Our findings suggest that it may be difficult for children to meet the recommendations for iodine as they get older due to increasing requirements.

Those with CG should, therefore, have an assessment of iodine intake as part of their routine dietetic review. Assessment for goitre and other signs and symptoms of hypothyroidism should be considered in those with insufficient iodine intake in conjunction with the medical team along with biochemical monitoring of thyroid function, as clinically indicated. Intake of iodine-fortified dairy alternative milk, white fish, eggs and permitted cheeses should be encouraged. The goitrogenic effect of soya versus other dairy alternatives should also be considered as an additional factor. For those unable to achieve adequate intake from diet alone, a supplement containing iodine should be considered. This study strengthens the argument for the iodine fortification of all dairy alternative products such as milk, yoghurt, custards and cheeses to the equivalent of dairy versions.

## Figures and Tables

**Table 1:** Iodine deficiency disorders according to physiological group adapted from [3].

Physiological Group	Health Consequences of Iodine Deficiency
All ages	Goitre Hypothyroidism
Foetus	Spontaneous abortion Stillbirth Congenital anomalies Perinatal mortality
Neonate	Endemic cretinism including mental deficiency with a combination of mutism, spastic diplegia, squint, hypothyroidism and short stature Infant mortality
Child and adolescent	Impaired cognitive function Delayed physical development Iodine-induced hyperthyroidism ^#^
Adults	Impaired cognitive function Iodine-induced hyperthyroidism ^#^

^#^ May occur in chronic deficiency if acutely exposed to excess iodine.

**Table 2:** Sample demographic characteristics and significant differences between demographics and meeting EFSA AI iodine recommendations.

N = 43	n	%	Meeting Recommendations (n)		*p*-Value
			Yes	No	
*Sex*					0.71
Male	17	39.5	8	9	
Female	26	60.5	15	11	
*EFSA AI age groups*					0.06
7–11 months	5	11.7	5	0	
1–10 years	25	58.1	13	12	
11–18 years	13	30.2	5	8	
*Ethnicity/cultural group*					0.41
Irish	20	46.5	12	8	
Irish Traveller	22	51.2	10	12	
Any other white background	1	2.3	1	0	
*Education level of parents*					0.11
Primary	3	7.0	1	2	
Secondary (lower and upper secondary)	22	51.2	9	13	
Third level and postgraduate	18	41.9	13	5	

**Table 3:** Projected impact of strategy 1, the fortification of dairy alternative milk only, and strategy 2, the fortification of dairy alternative milk, yoghurts, custards/desserts and cheeses with iodine to levels described in methods based on current levels of consumption (µg/day) on meeting EFSA AI values.

Age Group	% Meeting EFSA AI Values		
	Current Intake	Fortification Strategy 1	Fortification Strategy 2
1–10 years	52	72	100
11–18 years	39	69	77
1–18 years	47	71	92

## Data Availability

The data presented in this study are available on request from the corresponding author.

## Supplementary Materials

The following supporting information can be downloaded at: https://www.mdpi.com/article/10.3390/nu15020407/s1, Document S1: iodine-specific food frequency questionnaire; Document S2: iodine-specific food atlas.
