# Supplementary material for: Assessment of Dietary Intake of Iodine and Risk of Iodine Deficiency in Children with Classical Galactosaemia on Dietary Treatment
Source: Nutrients. 2023 Jan 13;15(2):407. doi: 10.3390/nu15020407 (PMC9860822; doi:10.3390/nu15020407)
Supplement: Supplementary file 1 [file nutrients-15-00407-s001.zip › Document S1. iodine-specific food frequency questionnaire.pdf]

Demographic Questionnaire

Name ________________________________

Date ________________________________

What is the sex of your child?

☐ Male ☐ Female

What age is your child?

☐

What is your child's ethnic or cultural background (tick most appropriate box)?

A. White

1. ☐ Irish
2. ☐ Irish Traveller
3. ☐ Any other White background

B. Black or Black Irish

4. ☐ African
5. ☐ Any other Black background

C. Asian or Asian Irish

6. ☐ Chinese
7. ☐ Any other Asian background

D. Other including mixed background

8. ☐ Other, write in description

What is the highest level of education you the parent/guardian have completed?

	Please put a tick (✔) on every line			
	**Nutritional supplements**			
Is your child on any vitamin and mineral supplements?	Yes		No	
If yes, please give details below				
Brand (e.g. Sanatogen, Seven Seas)				
Dose (e.g. number of tablets per day) – do they take it every day?				
Does your child take kelp or seaweed supplements?	Yes		No	
If yes, please specify the brand and dose				

**Salt**									
Do you use iodised salt?		Yes				No			
**What is the average amount of your individual portion of iodised salt used daily?**									
Less than ¼ teaspoon		¼ teaspoon		½ teaspoon		1 teaspoon		More than 1 teaspoon	

**Foods**					**On average how often have they eaten the following in the last year?**											
**Fish/shellfish** *(Eaten at home or from restaurant/take away. Cooked any way or in meals like stir-fry, pasta or soup)*	**Never or less than once a month**		**Once a month**		**Once in 2 weeks**		**Once a week**		**2-3 times per week**		**4-6 times per week**		**Once a day**		**More than once a day**	
	**Iodine intake (µg)**	**Portion size (g)**														
**WHITE FISH** *(fresh or frozen)*																
**Cod** *(Baked, steamed, battered, breaded, fried or grilled)*																
**Pollock/Coley** *(Baked, steamed, battered, breaded, fried or grilled)*																

**Fish/shellfish** *(Eaten at home or from restaurant/take away. Cooked any way or in meals like stir-fry, pasta or soup)*	**Never or less than once a month**		**Once a month**		**Once in 2 weeks**		**Once a week**		**2-3 times per week**		**4-6 times per week**		**Once a day**		**More than once a day**	
	**Iodine intake (µg)**	**Portion size (g)**														
**Haddock** *(Baked, steamed, battered, breaded, fried or grilled)*																
**Plaice** *(Baked, steamed, battered, breaded, fried or grilled)*																
**Hake** *Baked, steamed, battered, breaded, fried or grilled)*																
**Fish finger/Fish dipper** *(Eaten at home or from restaurant/take away. Including when added to salads or meals)*																
**Fish cake** *(White fish or salmon)*																
**OILY FISH***)*																
**Tinned pink/red salmon**																
**Fresh salmon**																
**Smoked salmon**																
**Tinned tuna**																
**Fresh tuna**																
**Tinned anchovy**																
**Tinned sardines**																
**Mackerel**																
**Kippers**																

**Fish/shellfish** *(Eaten at home or from restaurant/take away. Cooked any way or in meals like stir-fry, pasta or soup)*	**Never or less than once a month**		**Once a month**		**Once in 2 weeks**		**Once a week**		**2-3 times per week**		**4-6 times per week**		**Once a day**		**More than once a day**	
	**Iodine intake (µg)**	**Portion size (g)**														
**SHELLFISH**																
**Prawns/scampi** *(Eaten at home or from restaurant/take away. Cooked any way including or in meals like stir-fry, pasta or soup)*																
**Crab/Crab meat** *(Eaten at home or from restaurant/take away. Eaten fresh or cooked or in meals)*																
**Mussels** *(Eaten at home or from restaurant/take away. Cooked any way or in meals like pasta or soup)*																
**Oysters OR Scallops** *(Fresh, cooked or tinned. Including in meals)*																
**Squid/calamari** *(Eaten at home or from restaurant/take away. Cooked any way including crumbed/ battered)*																
**Lobster**																

**Fish/shellfish** *(Eaten at home or from restaurant/take away. Cooked any way including crumbed/ battered or in meals like stir-fry, pasta or soup)*	**Never or less than once a month**		**Once a month**		**Once in 2 weeks**		**Once a week**		**2-3 times per week**		**4-6 times per week**		**Once a day**		**More than once a day**	
	**Iodine intake (µg)**	**Portion size (g)**														
**OTHER**																
**Fish paste/fish spread/ Fish pâté**																
**Fish pie**																

**Dairy alternative products**	
**Milk – What type of dairy alternative milk does your child drink? How much do they drink in one week?**	
**SMA wysoy**	
**Neocate**	
If yes, how do you make it up?	
**Soya**	
**Almond/Nut**	

Oat
**Pea**
**Coconut**
**Would your child ever consume dairy alternative milks outside the home? For eg in tea, hot chocolate etc**<br>*If so, how much and how often?*

Dairy alternative yogurts, custard, ice cream and desserts	Never or less than once a month		Once a month		Once in 2 weeks		Once a week		2-3 times per week		4-6 times per week		Once a day		More than once a day	
	Iodine intake (µg)	Portion size (g)														

Permitted cheeses	Never or less than once a month		Once a month		Once in 2 weeks		Once a week		2-3 times per week		4-6 times per week		Once a day		More than once a day	
	Iodine intake (µg)	Portion size (g)														
Comté, Emmental, Jarlsberg, Italian Parmesan, Grana Padano, Vintage/Extra Mature cheese																

Dairy alternative cheeses or other cheeses	Never or less than once a month		Once a month		Once in 2 weeks		Once a week		2-3 times per week		4-6 times per week		Once a day		More than once a day	
	Iodine intake (µg)	Portion size (g)														

Egg	
**How many eggs does your child eat per week?**<br>*What type of egg? Normal/duck*<br><br>*Do they eat the whole of the egg? Do they have a preference for the white or the yolk?*	

Mixed dishes (cheese/eggs)	Never or less than once a month		Once a month		Once in 2 weeks		Once a week		2-3 times per week		4-6 times per week		Once a day		More than once a day	
	Iodine intake (µg)	Portion size (g)														
**Cheese pizza**																
**Quiche** *(eggs and cheese)*																
**Fresh pasta**																

Other foods	Never or less than once a month		Once a month		Once in 2 weeks		Once a week		2-3 times per week		4-6 times per week		Once a day		More than once a day	
	Iodine intake (µg)	Portion size (g)														
**Sushi- with seaweed**																
**Seaweed** *(including crisps, dried and cooked in Asian style soups and meals)*																
**Cashew nuts** *(Roasted and salted)*																

Does your child ever consume food that would not be recommended for him/her in terms of galactosaemia?																
	Never or less than once/month		Once a month		Once in 2 weeks		Once a week		2-3 times per week		4-6 times per week		Once a day		More than once a day	
	Iodine intake (µg)	Portion size (g)														
Cow’s milk																
Dairy chocolate																
Milkshakes																
Dairy yogurt																
Ice cream																
