# Supplementary material for: Assessment of Dietary Intake of Iodine and Risk of Iodine Deficiency in Children with Classical Galactosaemia on Dietary Treatment
Source: Nutrients. 2023 Jan 13;15(2):407. doi: 10.3390/nu15020407 (PMC9860822; doi:10.3390/nu15020407)
Supplement: Supplementary file 1 [file nutrients-15-00407-s001.zip › Document S2. iodine-specific food atlas.pdf]

Seafood

Cod

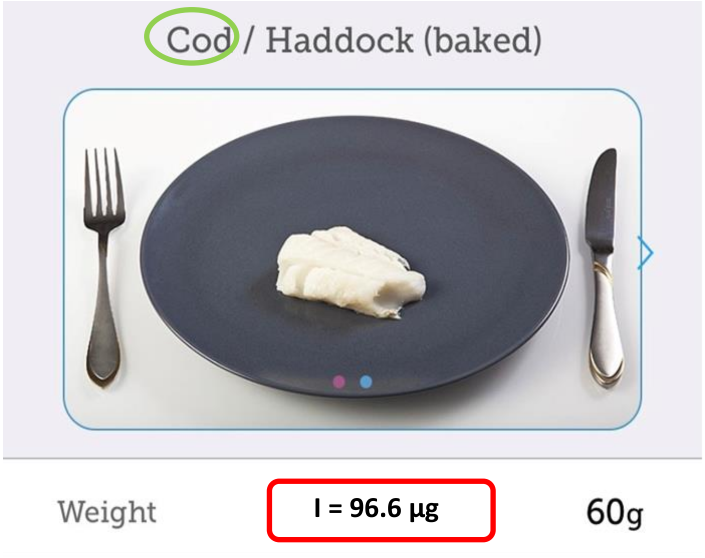

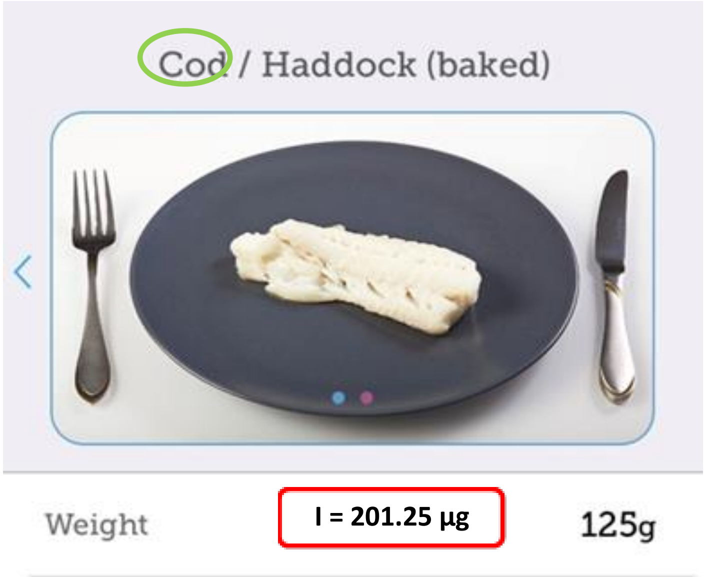

Cooking method, Cod	Iodine intake ug/100g	Iodine intake ug/60g	Iodine intake ug/125g
Cod baked	161	96.6	201.25
Cod, flesh only, grilled	276	165.6	345
Cod, flesh only, microwaved	243	145.8	303.75
Cod, flesh only, steamed	256	153.6	320
Cod, flesh only, salted and smoked, raw	110	66	137.5

Breaded Fish (Donegal catch, supermarket bought)

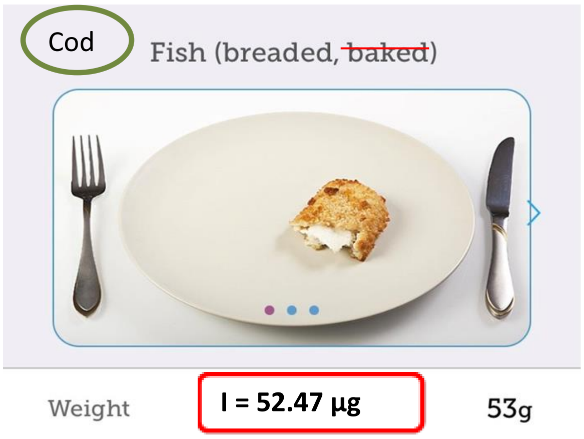

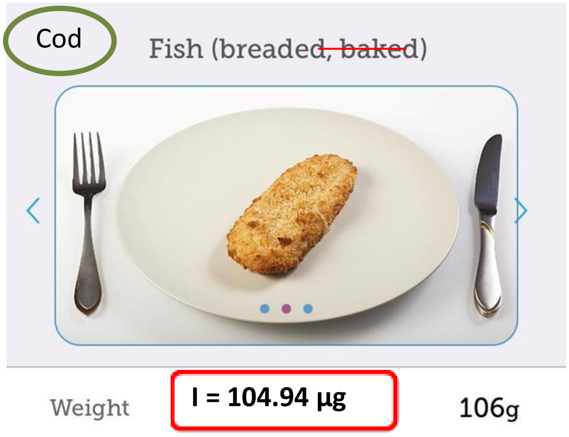

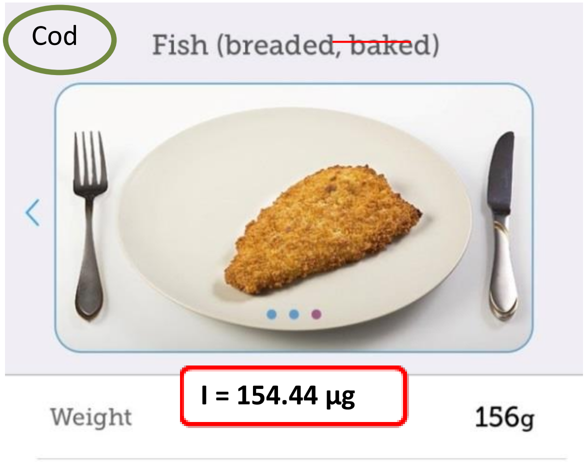

**Breaded fish**	**Iodine intake ug/100g**	**Iodine intake ug/53g**	**Iodine intake ug/106g**	**Iodine intake ug/156g**
Cod, in breadcrumbs, baked	99	52.47	104.94	154.44
Haddock, coated in crumbs, frozen, fried in blended oil	250	132.5	265	390
Plaice in breadcrumbs, baked	16	8.48	16.96	24.96

Fish from the takeaway

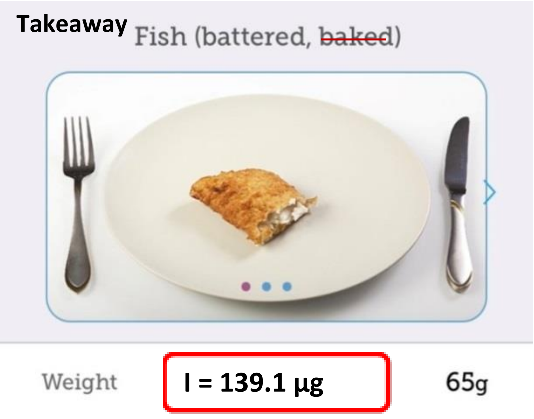

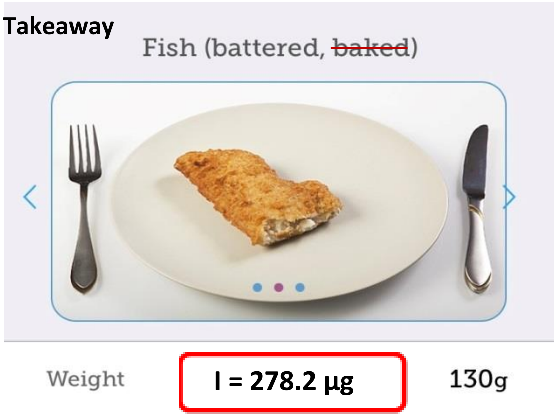

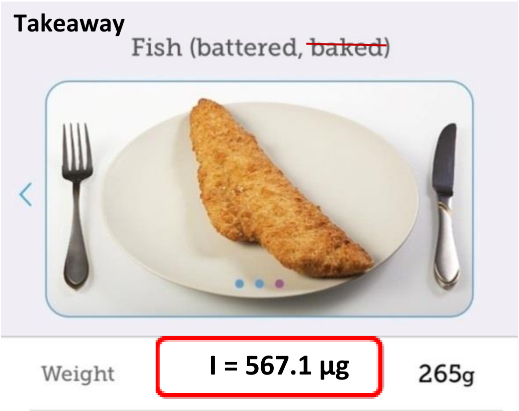

**Cod - takeaway**	**Iodine intake ug/100g**	**Iodine intake ug/65g**	**Iodine intake ug/130g**	**Iodine intake ug/265g**
Cod, in batter, fried in dripping, takeaway	214	139.1	278.2	567.1
Plaice, in batter, fried in retail blend oil	16	10.4	20.8	42.4

Fish cakes/fingers

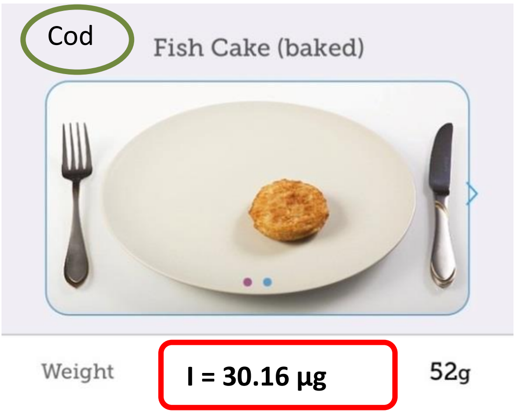

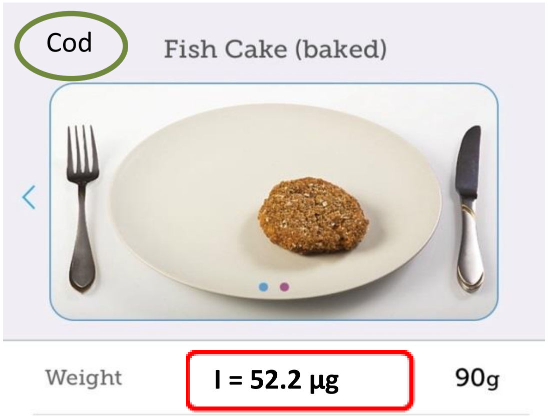

**Fish cake**	**Iodine intake ug/100g**	**Iodine intake ug/52g**	**Iodine intake ug/90g**
Fishcakes, *white fish*, coated in breadcrumbs, baked	58	30.16	52.2
Fishcakes, *salmon*, coated in breadcrumbs, baked	6	3.12	5.4

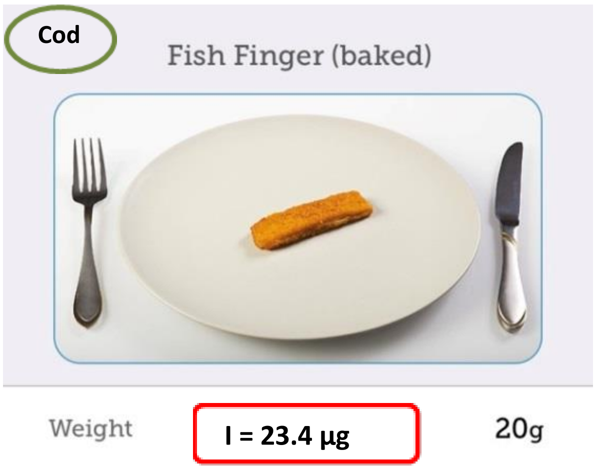

**Fish finger**	**Iodine intake ug/100g**	**Iodine intake ug/20g**
Fish fingers, *cod*, grilled/baked	117	23.4
Fish fingers, *cod*, fried in rapeseed oil	106	21.2
Fish fingers, *pollock*, grilled	47	9.4
Fish fingers, *salmon*, grilled/baked	6	1.2

Haddock/Pollock

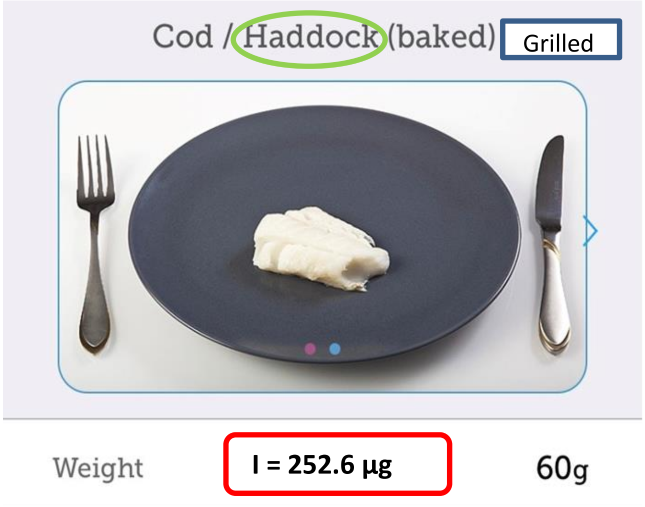

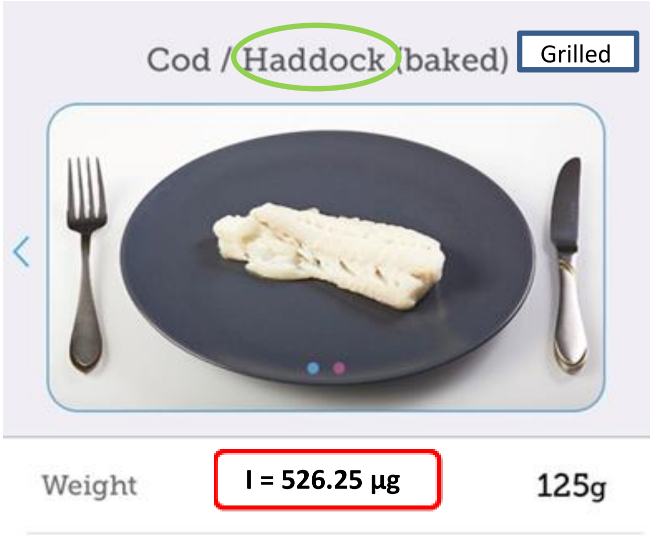

Cooking method, Cod	Iodine intake ug/100g	Iodine intake ug/60g	Iodine intake ug/125g
Haddock, flesh only, grilled	421	252.6	526.25
Haddock, flesh only, poached	409	245.4	511.25
Haddock, flesh only, steamed	381	228.6	476.25
Haddock, flesh only, smoked, steamed	257	154.2	321.25

Plaice

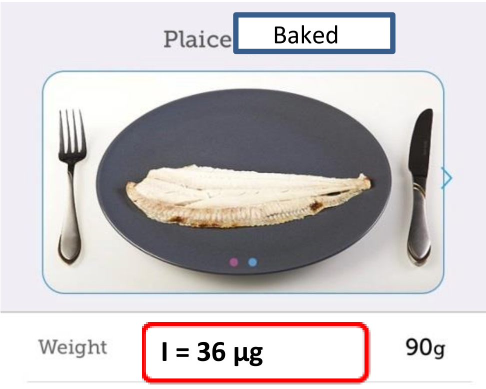

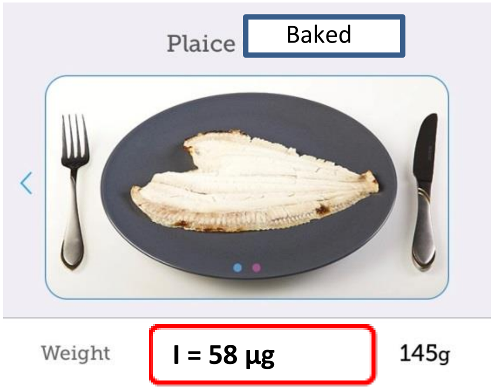

Cooking method, Plaice	Iodine intake ug/100g	Iodine intake ug/90g	Iodine intake ug/145g
Plaice, flesh only, baked	40	36	58
Plaice, flesh only, steamed	37	33.3	53.65

Salmon

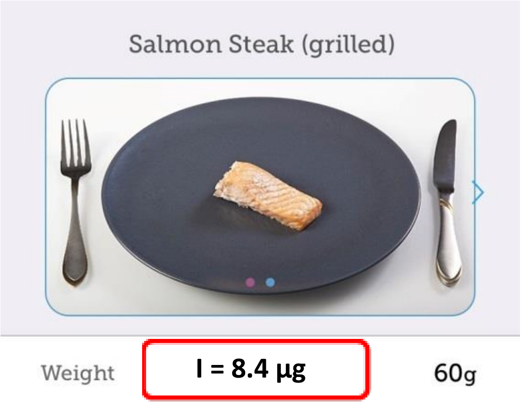

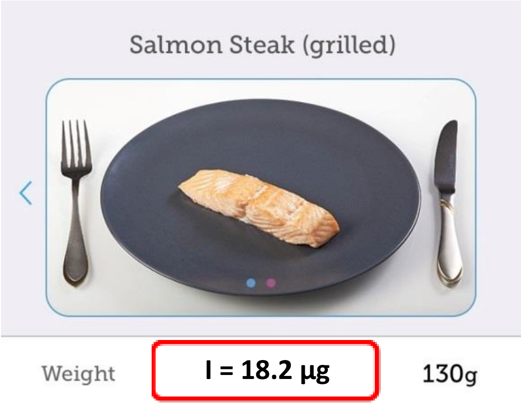

**Cooking method, Salmon**	**Iodine intake ug/100g**	**Iodine intake ug/60g**	**Iodine intake ug/130g**
Salmon*, farmed,* flesh only, grilled/baked/steamed	14	8.4	18.2
Salmon, *wild*, baked	15	9	19.5
Salmon, *wild,* grilled/steamed	14	8.4	18.2

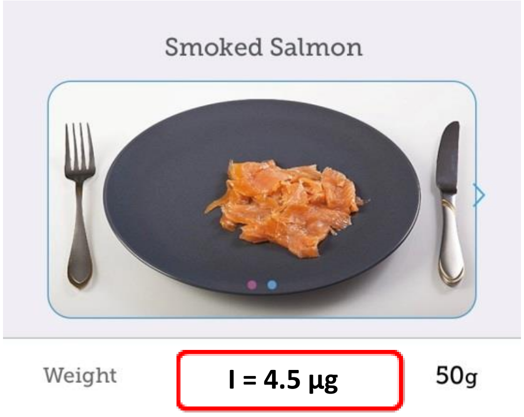

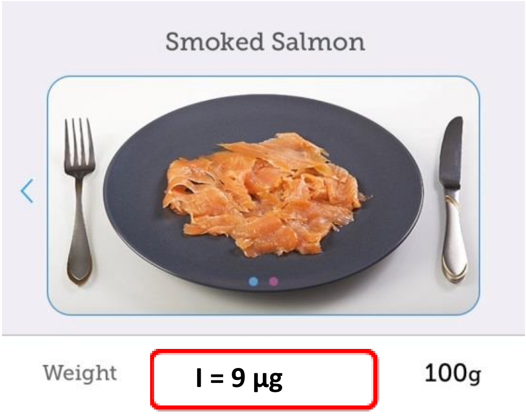

**Smoked salmon**	**Iodine intake ug/50g**	**Iodine intake ug/100g**
Salmon, smoked (cold/hot-smoked)	4.5	9

Tinned Salmon

RED Salmon (tinned in brine)

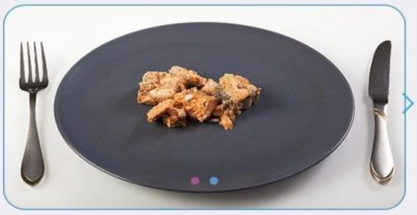

half tin

Weight **I = 20.4 µg** 85g

RED Salmon (tinned in brine)

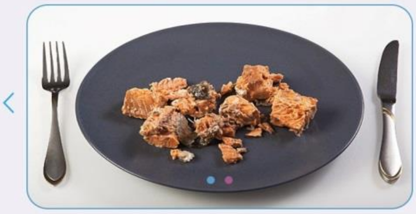

full tin

Weight **I = 40.8 µg** 170g

PINK Salmon (tinned in brine)

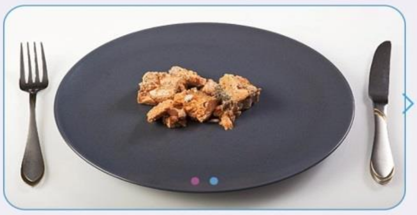

half tin

Weight **I = 15.3 µg** 85g

PINK Salmon (tinned in brine)

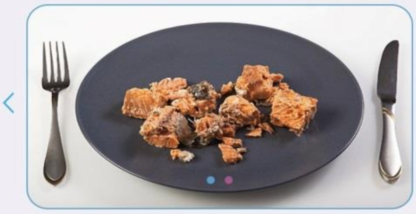

full tin

Weight **I = 30.6 µg** 170g

**Canned salmon**	**Iodine intake ug/100g**	**Iodine intake ug/85g**	**Iodine intake ug/170g**
Salmon, red, canned in brine, skinless and boneless, drained	24	20.4	40.8
Salmon, pink, canned in brine, drained	18	15.3	30.6

Tuna

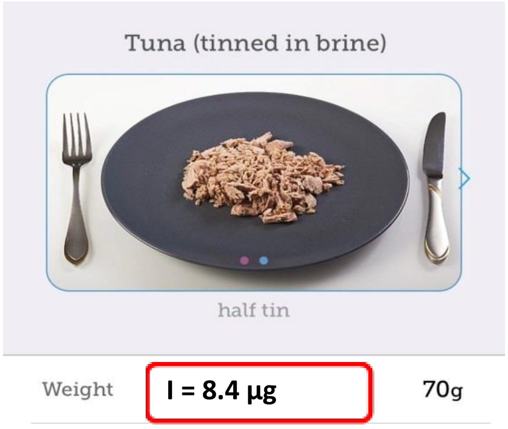

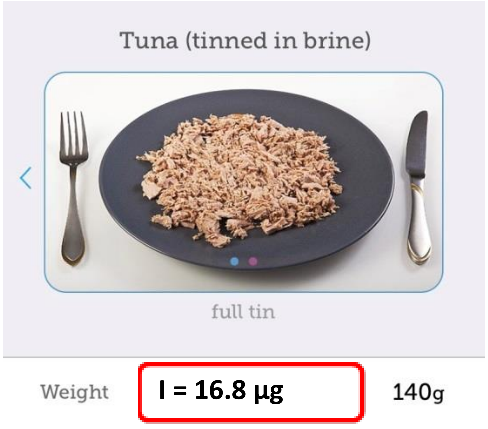

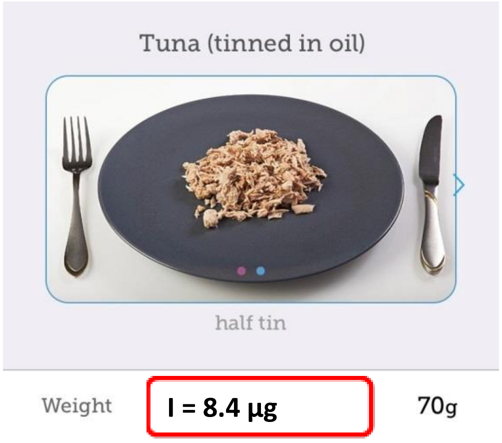

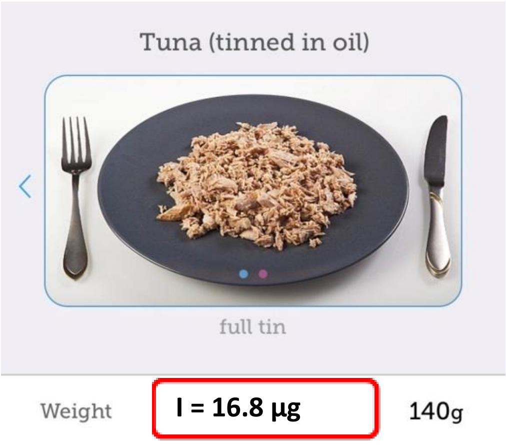

Tuna	Iodine intake ug/100g	Iodine intake ug/70g	Iodine intake ug/140g
Tuna, canned in brine, drained	12	8.4	16.8
Tuna, canned in sunflower oil, drained	12	8.4	16.8

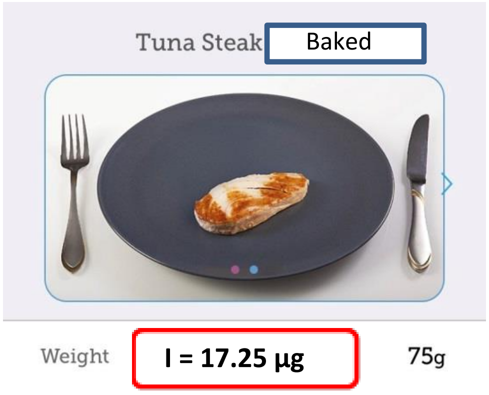

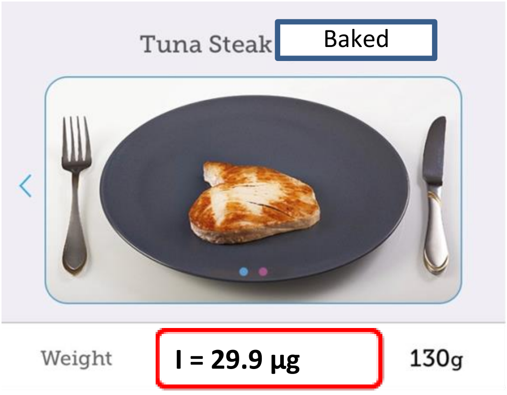

**Tuna**	**Iodine intake ug/100g**	**Iodine intake ug/75g**	**Iodine intake ug/130g**
Tuna, flesh only, baked	23	17.25	29.9

Sardines

I = 13 µg

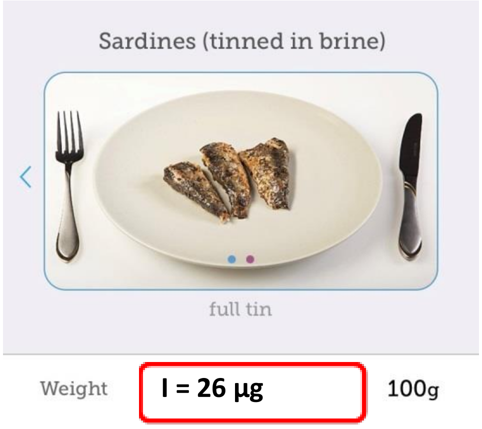

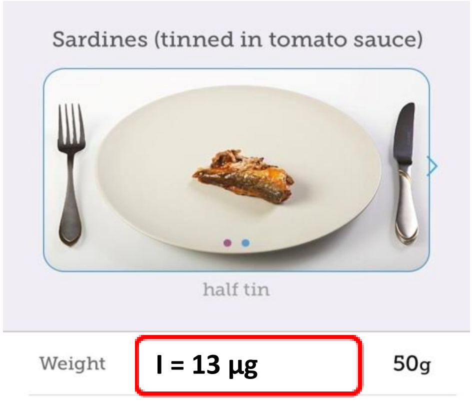

I = 26 µg

I = 11.5 µg

I = 23 µg

Sardines	Iodine intake ug/50g	Iodine intake ug/100g
Sardines, canned in brine, drained / tomato sauce	13	26
Sardines, canned in olive/sunflower oil, drained	11.5	23

Mackerel

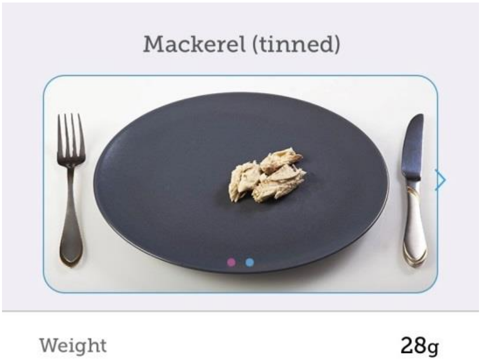

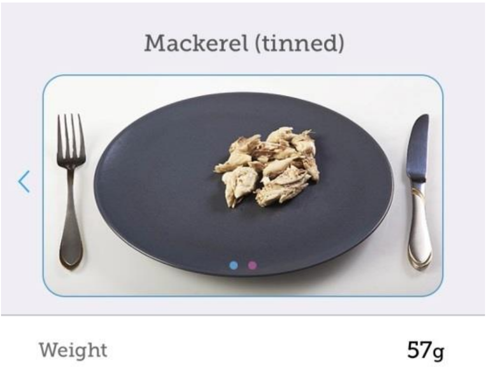

Mackerel	Iodine intake ug/100g	Iodine intake ug/28g	Iodine intake ug/57g
Mackerel, flesh only, grilled	35	9.8	19.95

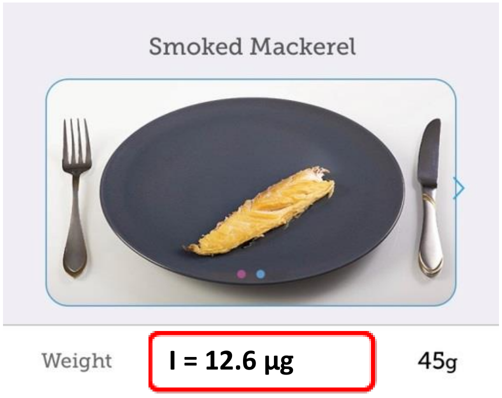

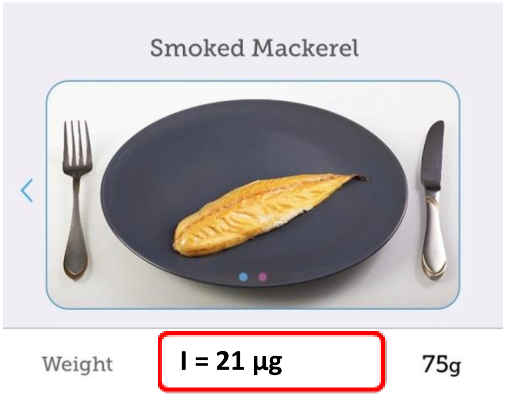

Mackerel	Iodine intake ug/100g	Iodine intake ug/45g	Iodine intake ug/75g
Mackerel, flesh only, smoked	28	12.6	21
Mackerel, flesh only, grilled	35	15.75	26.25

Prawns

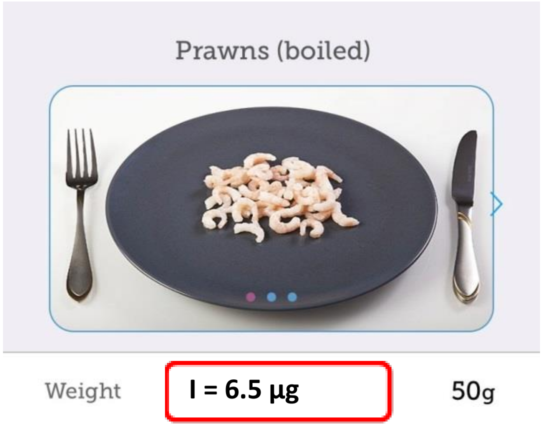

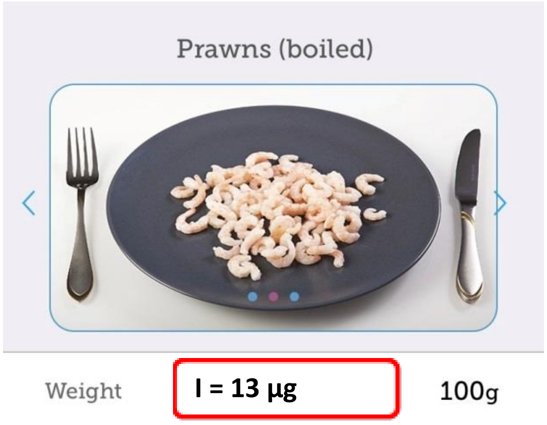

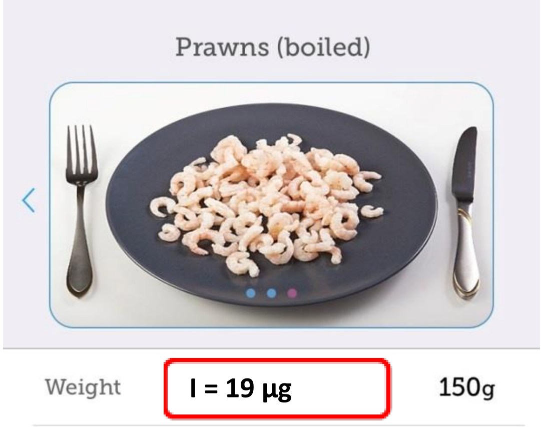

**Prawns**	**Iodine intake ug/50g**	**Iodine intake ug/100g**	**Iodine intake ug/150g**
Prawns, standard, purchased cooked	6.5	13	19

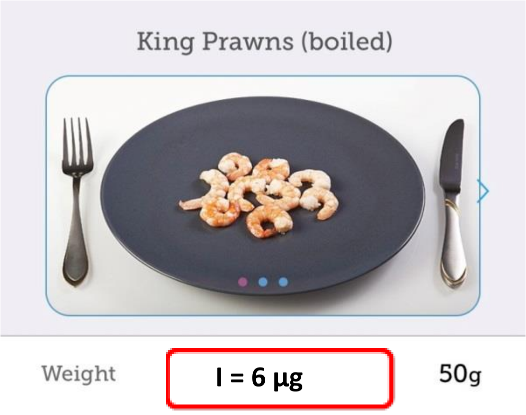

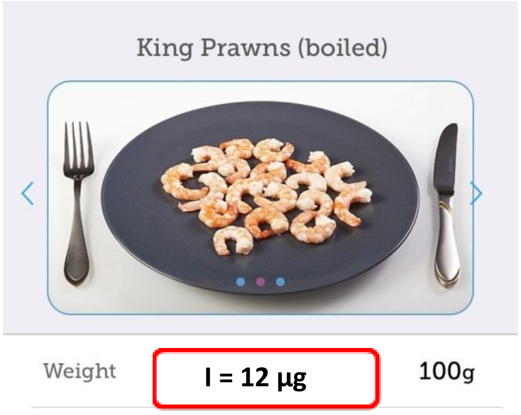

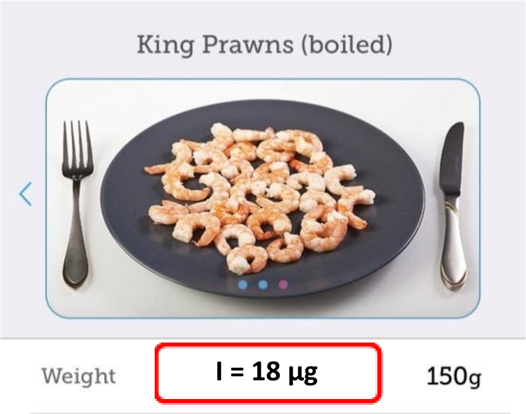

**Prawns**	**Iodine intake ug/50g**	**Iodine intake ug/100g**	**Iodine intake ug/150g**
Prawns, king, grilled from raw	3.5	7	10.5
Prawns, king, purchased cooked	6	12	18

Scampi

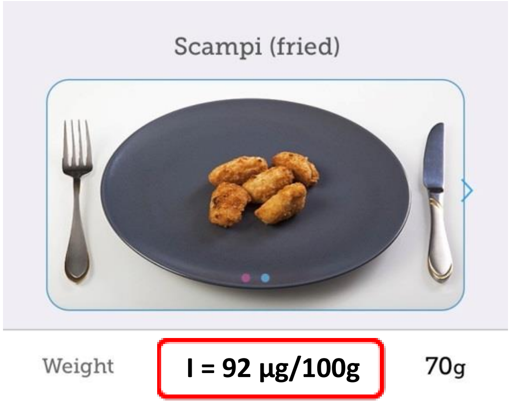

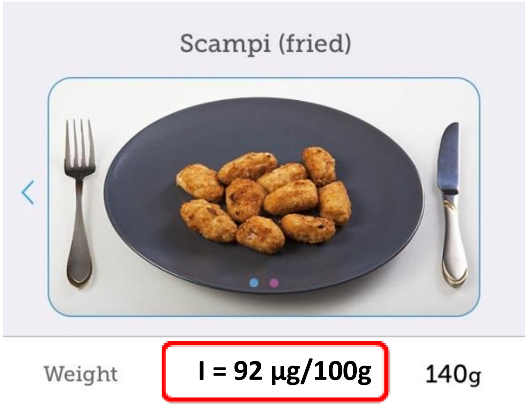

Scampi	**Iodine intake ug/100g**	**Iodine intake ug/70g**	**Iodine intake ug/140g**
Scampi, coated in breadcrumbs, baked	101	70.7	141.4
Scampi, coated in breadcrumbs, fried in oil	92	64.4	128.8

Crab Meat

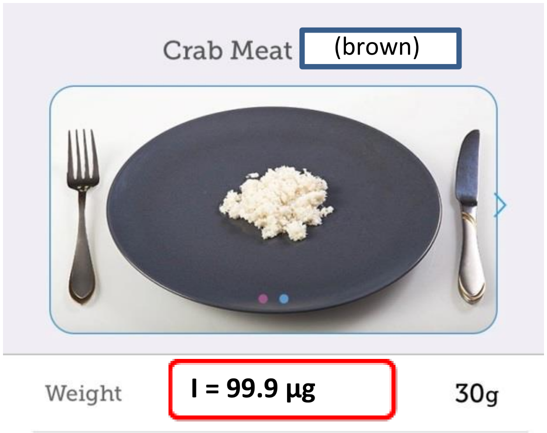

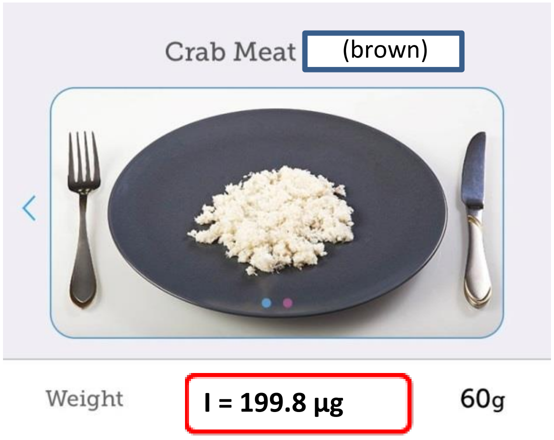

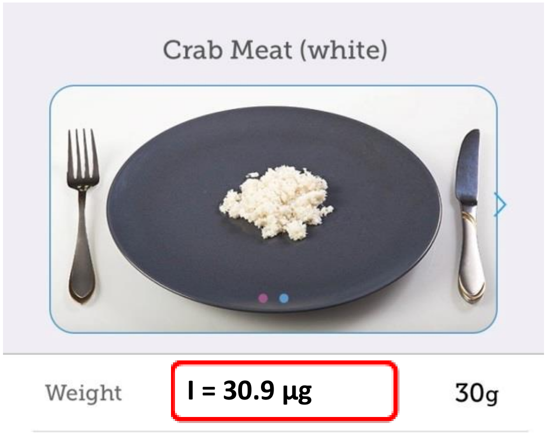

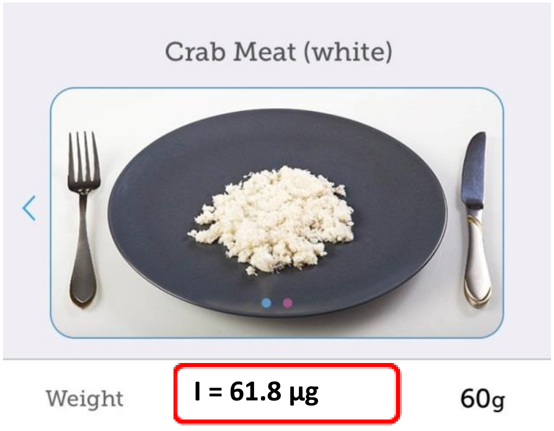

**Crab meat**	**Iodine intake ug/100g**	**Iodine intake ug/30g**	**Iodine intake ug/60g**
Crab, *brown meat*, purchased cooked	333	99.9	199.8
Crab, *white meat*, purchased cooked	103	30.9	61.8

Other

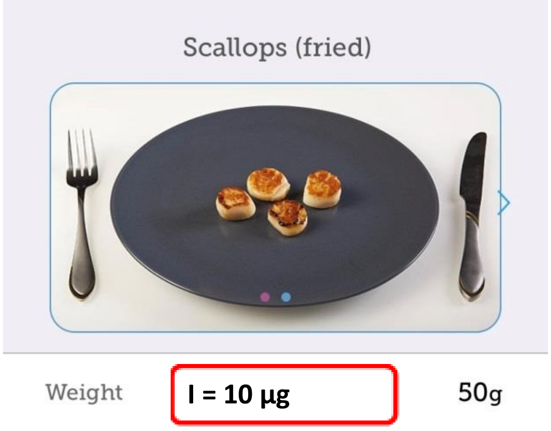

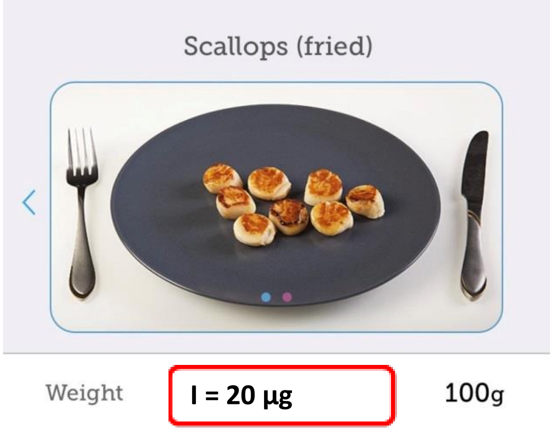

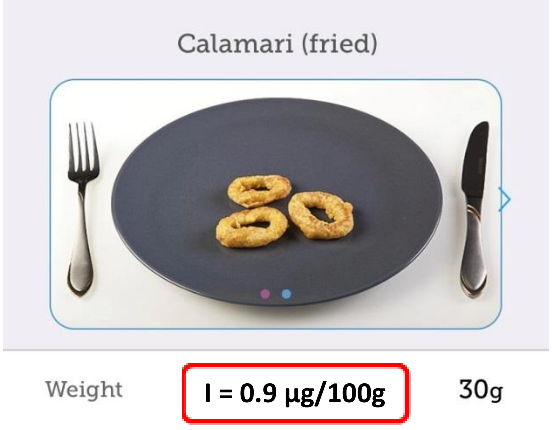

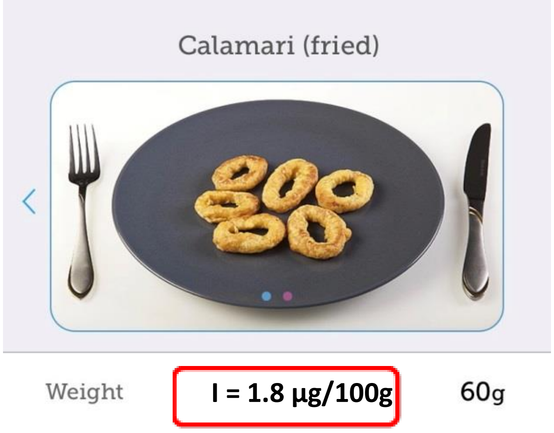

**Scallops**	**Iodine intake ug/50g**	**Iodine intake ug/100g**
Scallops, steamed	10	20

**Calamari**	**Iodine intake ug/100g**	**Iodine intake ug/30g**	**Iodine intake ug/60g**
Calamari, coated in batter, baked	3	0.9	1.8

Fish Pie

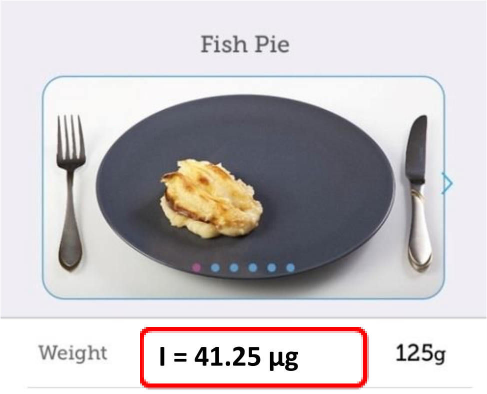

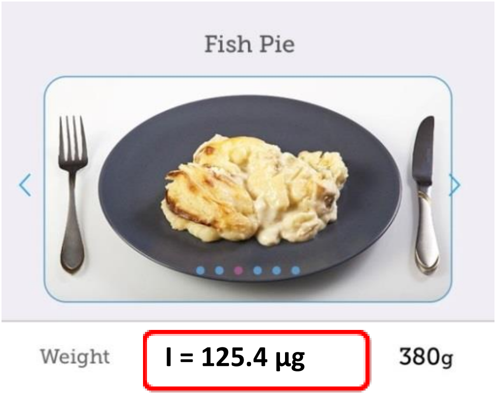

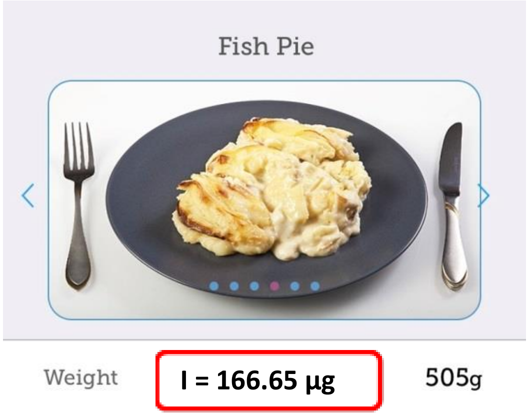

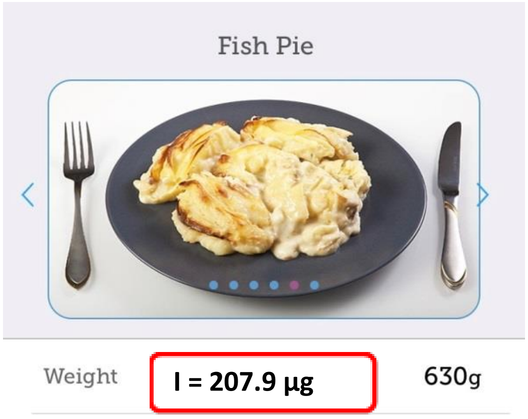

**Fish pie**	**Iodine intake ug/100g**	**Iodine intake ug/125g**	**Iodine intake ug/380g**	**Iodine intake ug/505g**	**Iodine intake ug/630g**
Pie, fish, *white fish*	33	41.25	125.4	166.65	207.9

Mussels

247 ug/100g

- Each pound of mussels (500g) = 125g mussel meat = roughly 20-25 mussels – *http://bestofsea.com/wp-content/uploads/2015/11/Mussels_Guide_Spreads_P9.pdf*

Dairy Alternative products

Milks

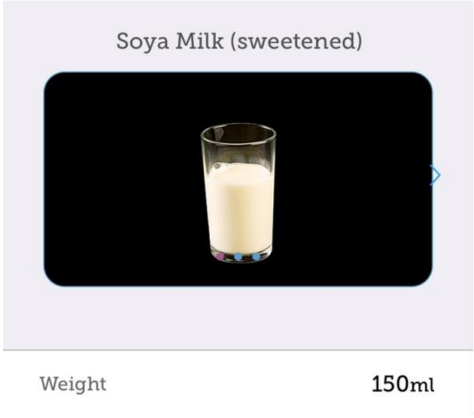

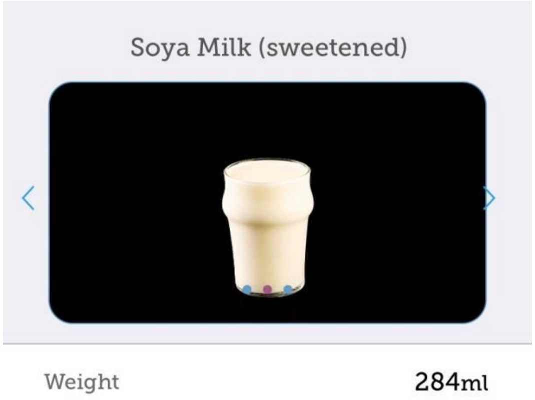

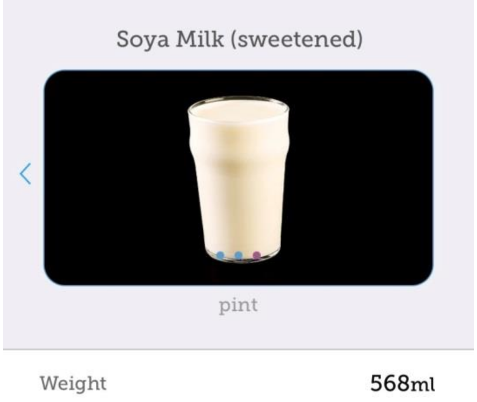

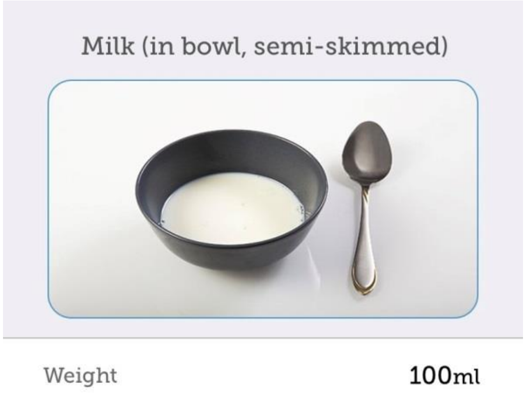

Iodine fortified Plant based Milks	Iodine content per 100ml
Alpro Soya Growing Up Drink 1-3+	24
Alpro Barista Soya	42
Koko Super Milk (UHT and Chilled)	13
M&S Plant Kitchen Soya Sweetened	45
M&S Plant Kitchen Soya Unsweetened	45
M&S Plant Kitchen Almond Sweetened	45
M&S Plant Kitchen Almond Unsweetened	45
M&S Plant Kitchen Coconut	30
M&S Plant Kitchen Oat	30
M&S Plant Kitchen Chocolate Oat	23
Mighty Pea Original	31
Mighty Pea Unsweetened	31
Oatly Classic Calcium	22.5
Oatly Barista Foamable	22.5
Oatly Chocolate	225

Milk outside the home

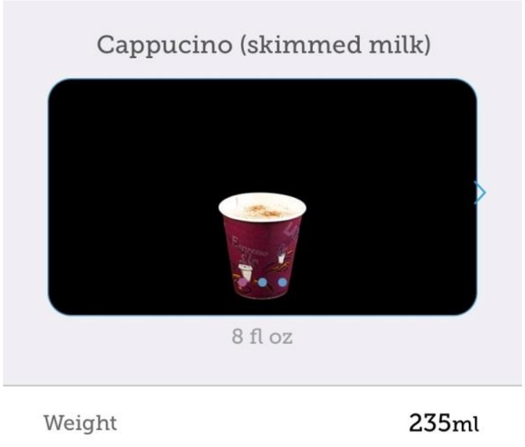

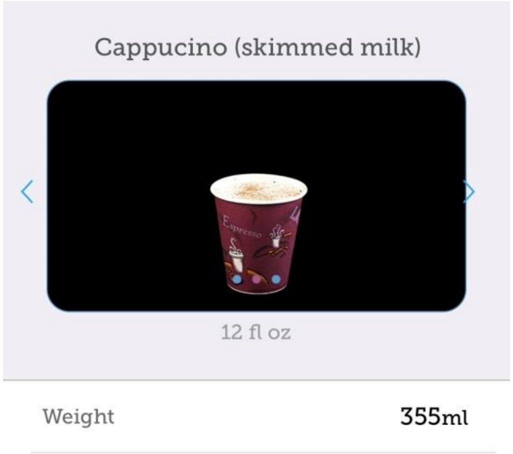

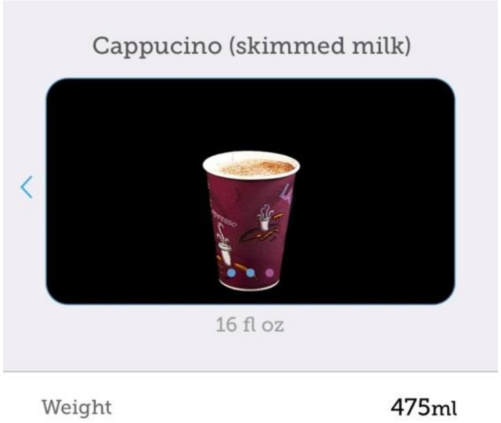

**Iodine fortified Plant based Milks**	**Iodine content per 100ml**	**Iodine content per 1L**
Alpro Soya Growing Up Drink 1-3+	24	240
Alpro Barista Soya	42	420
Koko Super Milk (UHT and Chilled)	13	130
M&S Plant Kitchen Soya Sweetened	45	450
M&S Plant Kitchen Soya Unsweetened	45	450
M&S Plant Kitchen Almond Sweetened	45	450
M&S Plant Kitchen Almond Unsweetened	45	450
M&S Plant Kitchen Coconut	30	300
M&S Plant Kitchen Oat	30	300
M&S Plant Kitchen Chocolate Oat	23	230
Mighty Pea Original	31	310

Mighty Pea Unsweetened	31	310
Oatly Classic Calcium	22.5	225
Oatly Barista Foamable	22.5	225
Oatly Chocolate	225	225

Yogurt

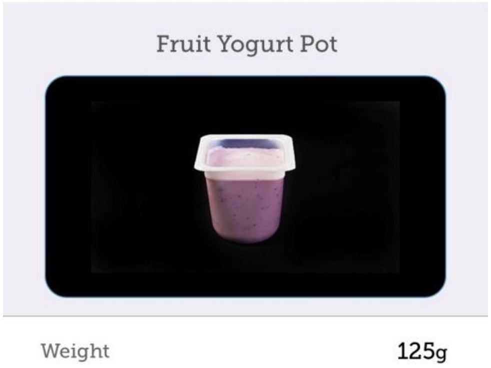

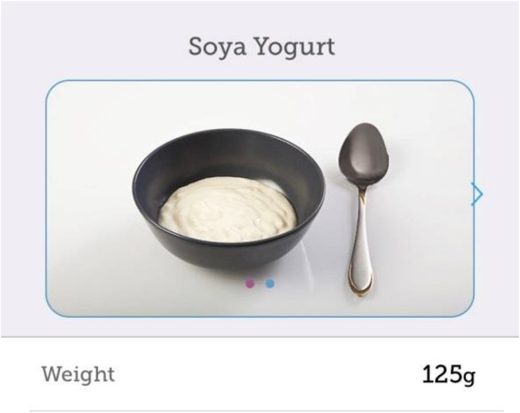

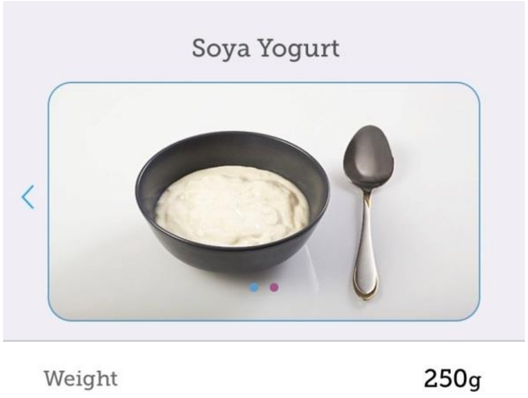

Permitted cheeses

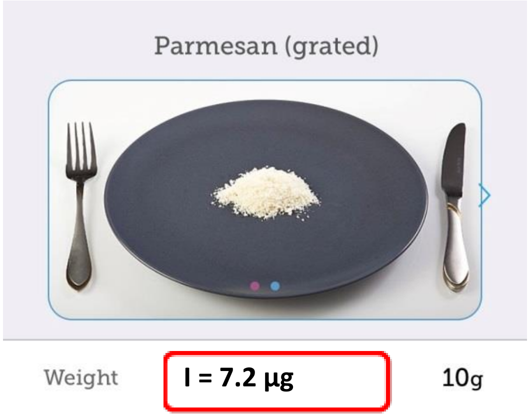

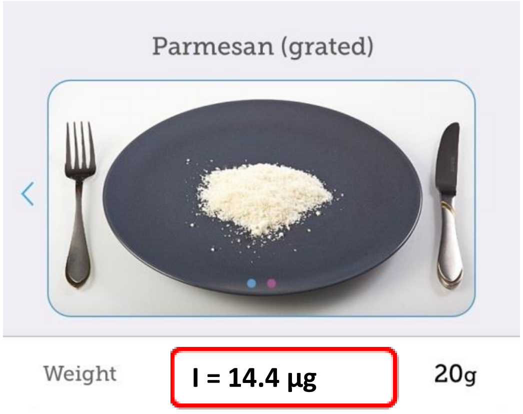

Parmesan	Iodine intake ug/100g	Iodine intake ug/10g	Iodine intake ug/20g
Parmesan	72	7.2	14.4

Slices/Grated/Block

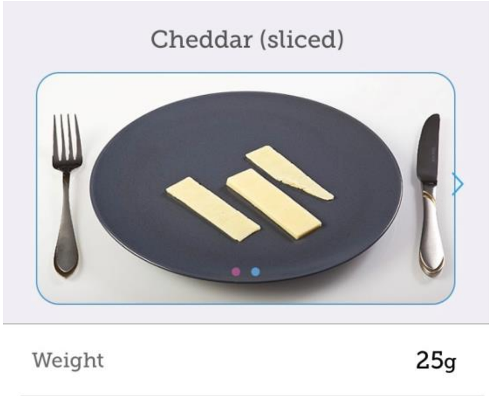

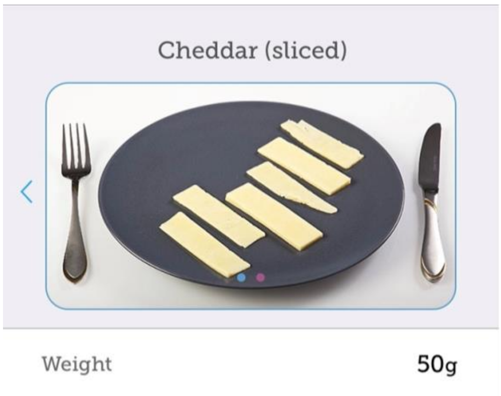

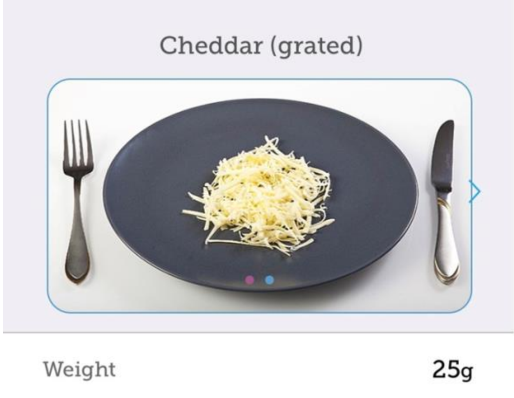

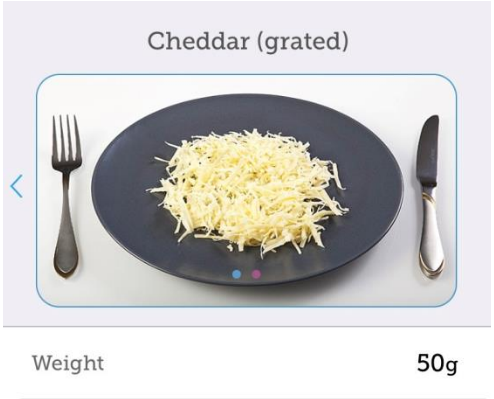

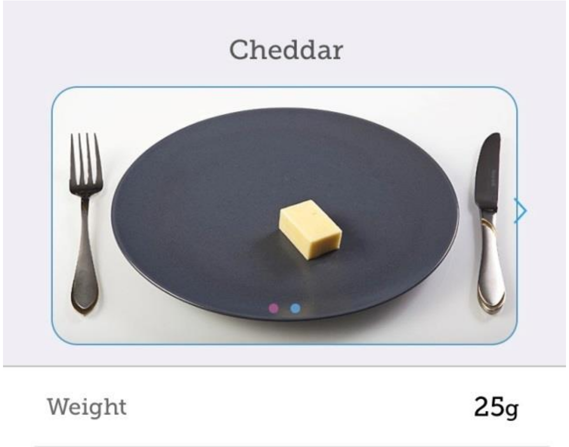

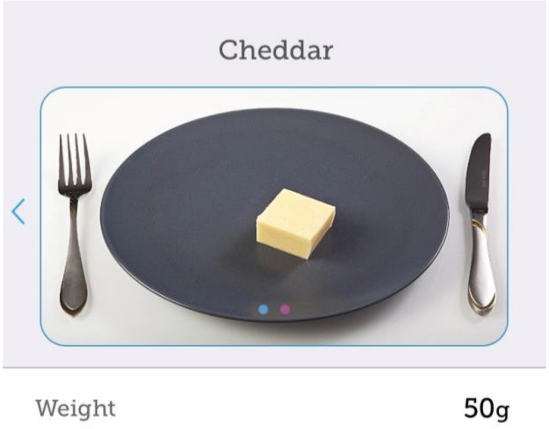

**Permitted Cheese**	**Iodine intake ug/100g**	**Iodine intake ug/25g**	**Iodine intake ug/50g**
Emmental	18	4.5	9
Cheese, hard, average	41	10.25	20.5
Jarlsberg			
Comte			
Gruyere			
**Other Cheeses**			
Cheese, Brie, with outer rind removed	16	4	8
Cheese, Cheddar, English	30	7.5	15
Cheese, Halloumi	60	15	30
Cheese, Mozzarella, fresh	18	4.5	9
Cheese, processed, plain	27	6.75	13.5
Cheese, spreadable, full fat, soft, white	11	2.75	5.5

Other foods

Fresh pasta – 36 µg/100g

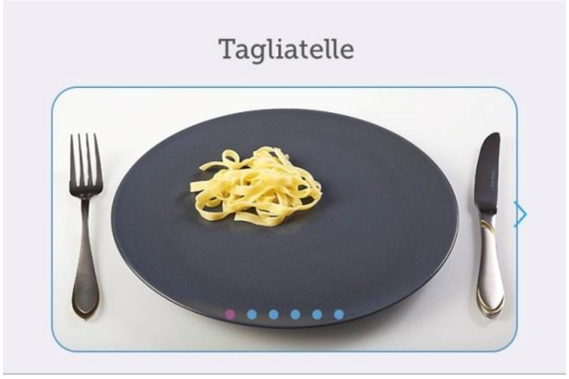

Weight | I = 10.8 µg | 30g

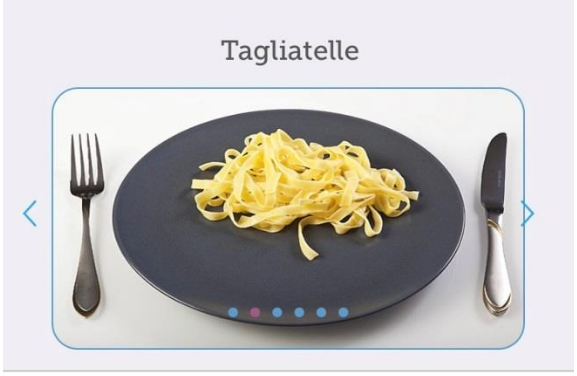

Weight | I = 32.4 µg | 90g

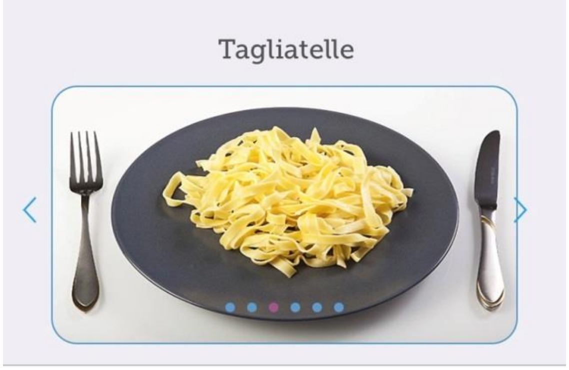

Weight | I = 54 µg | 150g

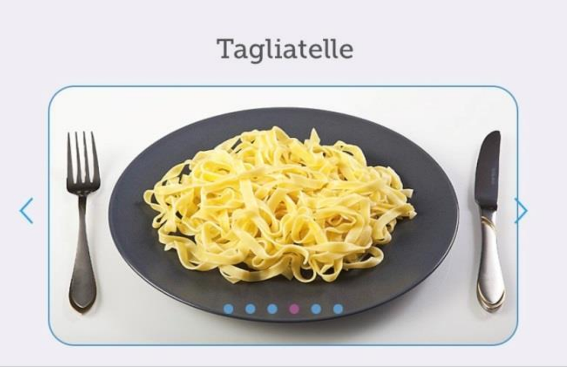

Weight | I = 75.6 µg | 210g

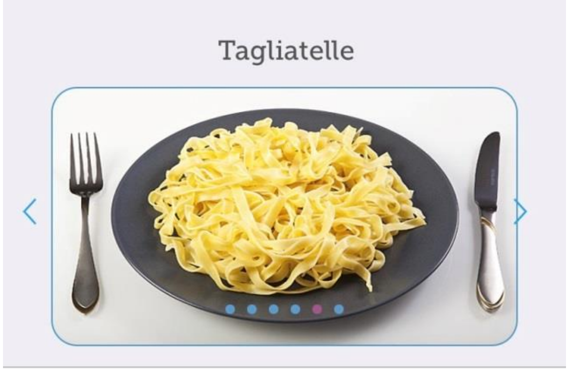

Weight | I = 97.2 µg | 270g

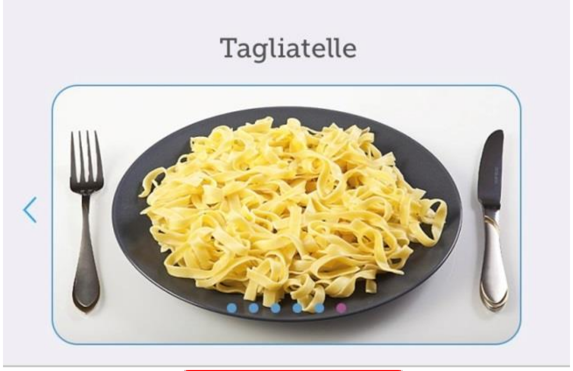

Weight | I = 118.8 µg | 330g

Eggs - 58 µg/100g

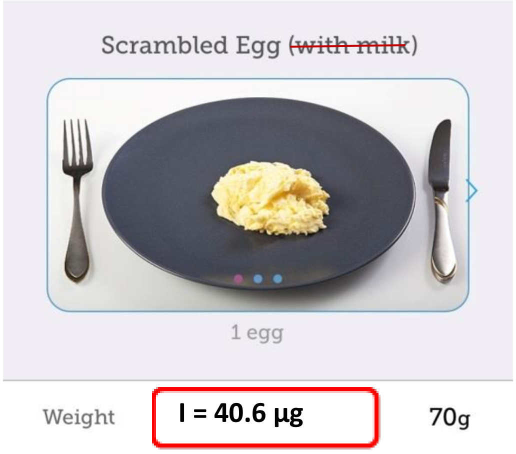

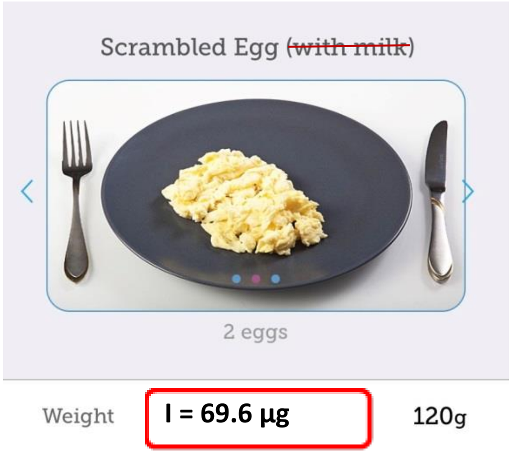

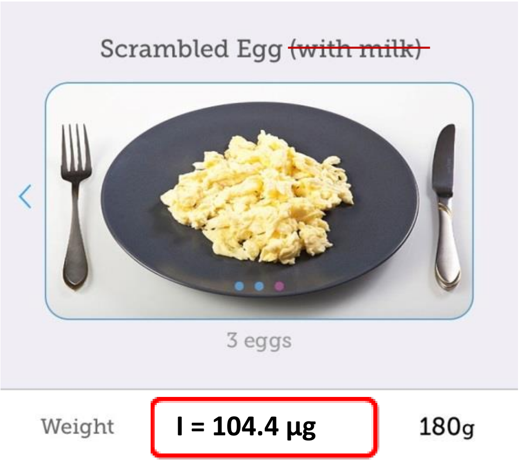

Eggs	Iodine intake ug/70g	Iodine intake ug/120g	Iodine intake ug/180g
Eggs, chicken, whole, scrambled, without milk	40.6	69.6	104.4

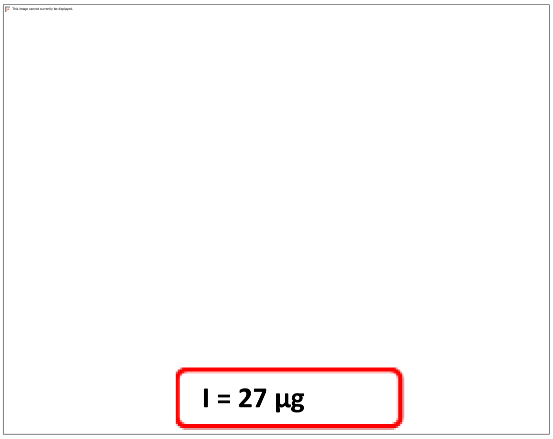

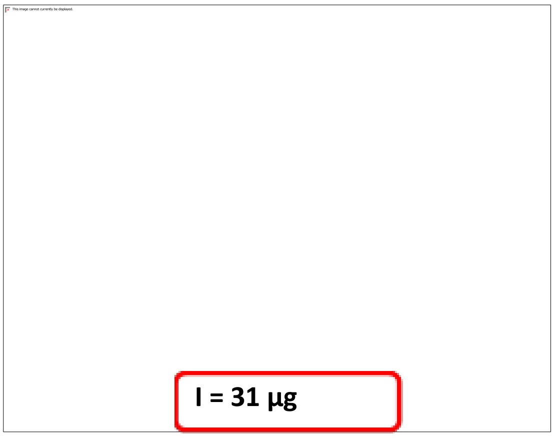

**Eggs**	**Iodine intake ug/100g**
Eggs, chicken, whole, poached	54
Eggs, chicken, whole, boiled	52

Other foods

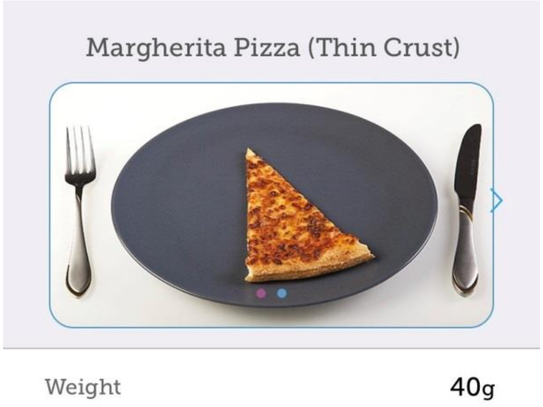

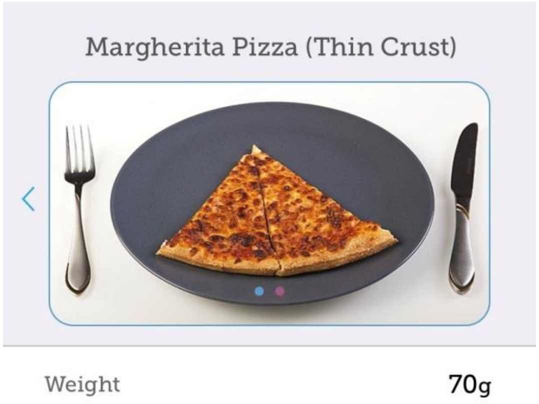

Pizza deep pan/thin crust slices	Type of Pizza	Weight of cheese (g)	Emmental Iodine intake	Cheese, hard, average Iodine intake	Cheese, Port Salut, St Paulin type Iodine intake	Cheese, Mozzarella, fresh Iodine intake
5” diameter	Average, cheese & tomato	116	20.88	47.56	27.84	20.88
	Meat/fish	160	28.8	65.6	38.4	28.8
	Vegetarian	150	27	61.5	36	27
	‘Special’	190	34.2	77.9		34.2
6” diameter children’s	Average, cheese & tomato	220	39.6	90.2	45.6	39.6
7” diameter (serves 1)	Average, cheese & tomato	230	41.4	94.3	55.2	41.4
	Meat/fish	29	5.22	11.89	6.96	5.22
	Vegetarian	300	54	123	72	54
	‘Special’	360	64.8	147.6	86.4	64.8
1 slice = ¼ of total weight of pizza						
9-10” diameter (serves 1-2)	Average, cheese & tomato	410	73.8	168.1	98.4	73.8
	Meat/fish	500	90	205	49.2	90
	Vegetarian	500	90	205	49.2	90
	‘Special’	580	104.4	237.8	139.2	104.4
1 slice = 1/6th of total weight						

of pizza						
12” diameter (serves 2-3)	Average, cheese & tomato	700	287	287	168	287
	Meat/fish	800	144	328	192	144
	Vegetarian	800	328	328	192	328
	‘Special’	890	160.2	364.9	213.6	160.2
1 slice = 1/8th of total weight of pizza						

**Pizza thin crust slices**	**Type of Pizza**	**Weight of cheese (g)**	**Emmental Iodine intake**	**Cheese, hard, average Iodine intake**	**Cheese, Mozzarella, fresh Iodine intake**
7” diameter (serves 1)	Average, cheese & tomato	116	20.88	47.56	20.88
	Meat/fish	150	27	11.07	27
	Vegetarian	150	27	11.07	27
	‘Special’	190	34.2	77.9	34.2
1 slice = ¼ of total weight of pizza					
9-10” diameter (serves 1-2)	Average, cheese & tomato	260	46.8	106.6	46.8
	Meat/fish	310	55.8	127.1	55.8
	Vegetarian	340	61.2	139.4	61.2
	‘Special’	350	63	143.5	63
1 slice = 1/6th of total weight of pizza					
12” diameter (serves 2-3)	Average, cheese & tomato	560	163.8	229.6	163.8
	Meat/fish	660	118.8	500.2	118.8
	Vegetarian	660	118.8	500.2	118.8
	‘Special’	740	133.2	303.4	133.2
1 slice = 1/8th of total weight of pizza					

Type of Pizza	Size	Weight of cheese (g)	Emmental/Moz Iodine intake	Cheese, hard, average Iodine intake
Chilled/frozen	Large	350-500	63-90	143.5-205
	Medium	200	36	82
Frozen	Fun size	40	7.2	16.4
	Individual slices	100	18	41
	Small	100	18	41
Homemade	Average portion	300	54	123

Quiche

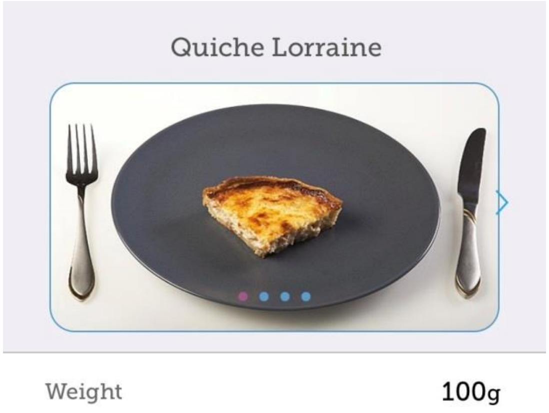

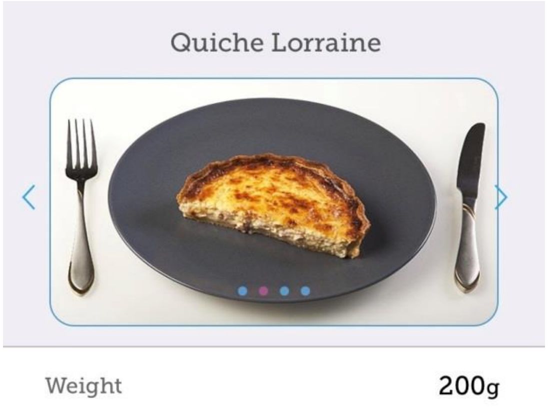

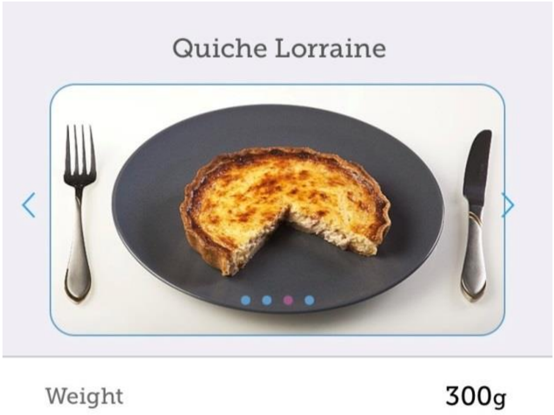

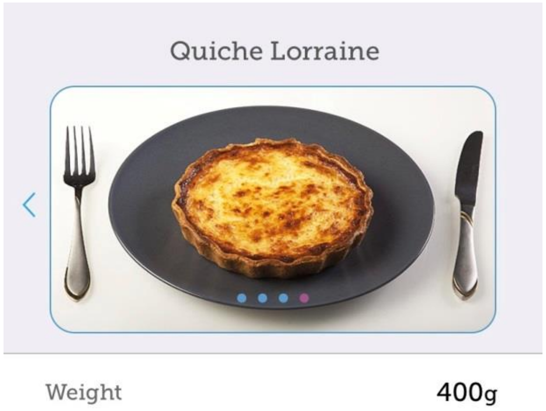

Iodised salt

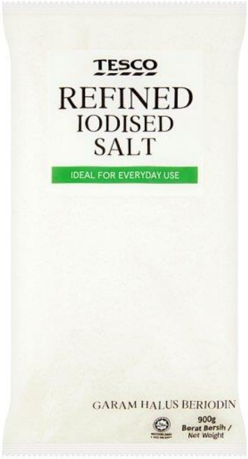

Sushi

Tuna Nigiri

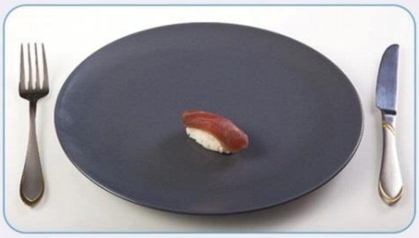

Weight I = 1.12 µg 28g

Tuna Sashimi

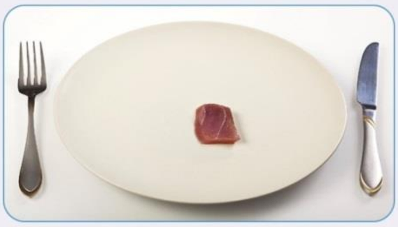

Weight I = 1.12 µg 15g

Salmon Nigiri

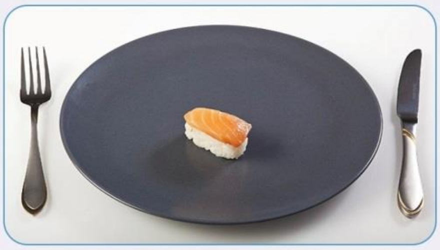

Weight I = 1.36 µg 34g

Salmon Sashimi

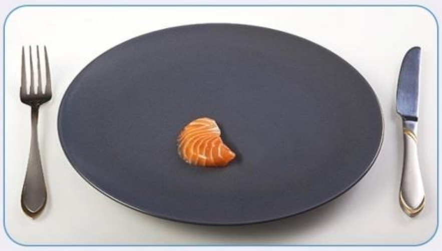

Weight I = 1.36 µg 15g

Mackerel Sashimi

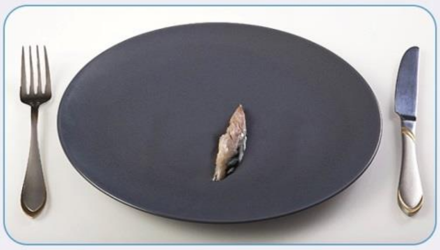

Weight I = 5.25 µg 15g

Cashew Nuts

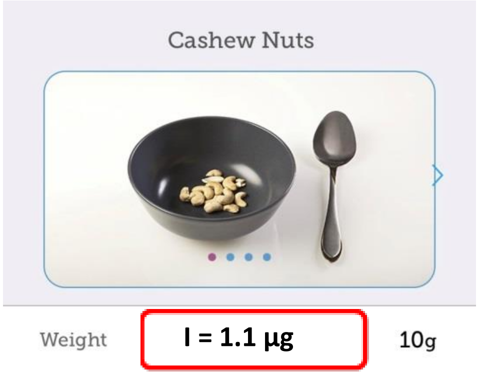
Cashew Nuts
Weight
I = 1.1 µg
10g

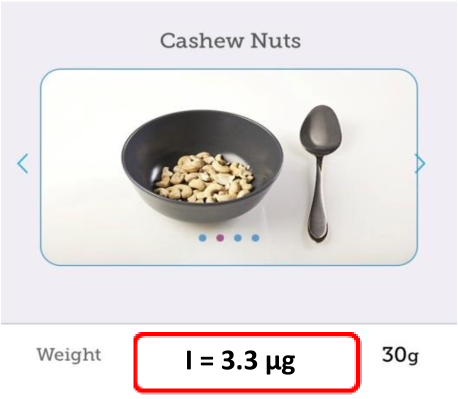
Cashew Nuts
Weight
I = 3.3 µg
30g

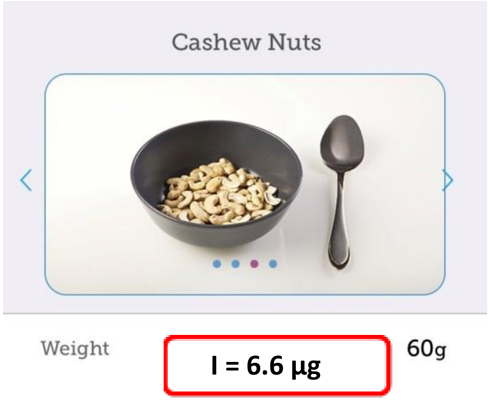
Cashew Nuts
Weight
I = 6.6 µg
60g

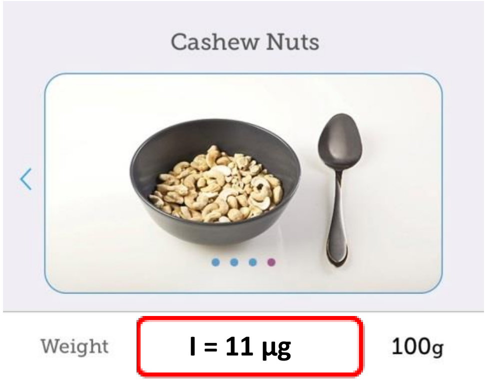
Cashew Nuts
Weight
I = 11 µg
100g
